# Adversity and cooperation in heterogeneous pairs

**DOI:** 10.1038/s41598-019-46624-8

**Published:** 2019-07-15

**Authors:** Kris De Jaegher

**Affiliations:** Utrecht University School of Economics, Utrecht University, Utrecht, The Netherlands

**Keywords:** Computational models, Evolutionary theory

## Abstract

This paper provides a game-theoretic model of the effect of higher adversity on the evolution of cooperation. The focus lies on how this effect of higher adversity is impacted when there is transient, non-genetic heterogeneity in the form of differences in the players’ capabilities of contributing to the public good, in the benefits they obtain from the public good, or in their cooperation costs. A framework is provided that identifies the common mechanisms that are at work across two models of cooperation (jointly producing a public good, and jointly defending an existing public good), and across the mentioned types of heterogeneity. With relatively small heterogeneity, higher adversity generates a common-enemy effect for large cooperation costs and a deterrence effect for small cooperation costs. Yet, these results on the effect of higher adversity are completely reversed for relatively large heterogeneity.

## Introduction

Explaining the evolution of cooperation is one of the key themes in evolutionary biology^1–7^. Standard explanations for the evolution of cooperation include direct^8^ and indirect reciprocity^9^, kin selection^10^, group selection^11^, and network reciprocity^12^. An alternative explanation is by-product mutualism, which argues that organisms cooperate whenever it is in their individual interests to do so^13,14^. Specifically, following an argument that dates back to Kropotkin^15^, the common-enemy hypothesis of by-product mutualism argues that cooperation arises when organisms face the “common enemy of a sufficiently adverse environment” [^16^, p.273], where the common enemy may be biotic (e.g., a predator) or abiotic (e.g., climatic conditions). This hypothesis thus provides a rationale for the phenotypic plasticity of cooperative behaviour^17^, which would be triggered by harsh environments.

At the same time, explanations of the evolution of cooperation often only model homogeneous organisms, whereas cooperating organisms may typically be heterogeneous^18–21^. Part of the literature on reciprocity^22–24^, on group selection^18^, and on network reciprocity^25–29^ has already shown that heterogeneity can fundamentally affect predictions on the evolution of cooperation. Also, part of the literature on kin selection can be interpreted as investigating how heterogeneity mediates the effect of relatedness on cooperation^30^. An analysis of heterogeneity in the context of the common-enemy hypothesis is so far missing, and this report attempts to fill this gap. As will be shown, analysing the effect of heterogeneity in this context is important, as sufficiently large heterogeneity is predicted to fundamentally change the incidence of the common-enemy effect.

We focus on transient, non-genetic heterogeneity, where because of random environmental conditions, players differ according to their ability to contribute to the public good, their costs of contributing to the public good, or the benefits they obtain from the public good (this form of heterogeneity has also been referred to as ecological asymmetry^31^; a related concept is the one of social diversity^25^, defined as differences in the scaling factor relating payoffs to fitness). Such a focus is justified, first, simply because of the importance of transient heterogeneity (see^23^); second, because even if heterogeneous traits are non-transient and subject to evolution, these traits may evolve for reasons unrelated to the cooperative situation considered ([^32^, p.160]; third, because even if heterogeneous traits evolve directly as a result of the cooperative situation, then as a first step one still needs to know how cooperative behaviour evolves, once evolution has led to heterogeneous groups being formed.

The common-enemy hypothesis has been formulated in several disciplines^33–37^, including evolutionary biology^16,38–42^. Most accounts in evolutionary biology combine the hypothesis with standard explanations of cooperation. For instance, when higher adversity takes the form of larger cooperation costs, these costs in the short run wipe out cooperators who are matched with defectors; yet, in case of positive assortment this short-run effect may actually promote cooperation in the long run^43^ (several other treatments combine harsh environments with forms of network reciprocity^25,44–48^). Another account proposes that higher adversity in the form of a higher risk of predation can give potentially cooperating prey more reasons to repeatedly interact, and may thus promote reciprocity^49^. Finally, the common-enemy has been modeled as taking the form of a competing group, in a theory that includes elements of group selection^50^.

Yet, in order to get insight in the mechanism underlying the common-enemy hypothesis, it is important to consider it separately from the standard rationales for cooperation. Two existing theories serve this purpose. In a first theory^51^, payoffs in two-player games take the form of survival probabilities. Adversity can be measured by the number of iterated attacks a pair of players faces, where an attack eliminates each player with a probability that depends on the number of cooperating players in the pair. In a second theory^52^, which is the focus of this report, cooperating in a pair means contributing to a public good, in two modelling variants. In the *production variant* cooperating means providing inputs for the production of a public good, and in the *defence variant* cooperating means investing in the defence of an existing public good. In both variants, adversity is reflected by the extent to which a second cooperating player in a pair contributes more than a first cooperating player, and thus by the extent to which each player’s contribution is pivotal. For instance, in cooperative hunting^53,54^, the larger the prey the more may be lost when a second hunting predator in a pair fails to hunt, and the less may be gained when a first predator starts hunting. Also, in territorial defence of a common territory^55–57^, the harsher the environment in the form of a larger number of attacks on the territory, the larger the reduction in the probability of keeping the territory if a single of two animals fails to defend, and the smaller the increase in the probability of keeping the territory if only one of two animals starts defending. Thus, while in the most general sense adversity means a reduction in the payoffs^27^, the argument here is that specifically in contexts where players contribute to a public good, higher adversity makes each player’s contribution to the public good more pivotal. It is into these modelling variants that we introduce heterogeneity.

## Model

In both modelling variants, at any point of time, the population consists of a fraction of strong and weak players (where strong players benefit more from cooperating than weak players). These fractions do not vary across time, and players are randomly assigned to the strong and weak roles independently of the roles they had in the past. Also, players are randomly matched in pairs independently from their roles (see the Methods section for the exact details). The cases of interest are subgames where a strong and a weak player are matched. Such subgames can be represented by the asymmetric bimatrix game in Table 1. The row player is the strong player (*s*), and the column player is the weak player (*w*). Each of the players can either contribute to a public good (cooperate, *C*), or not contribute (defect, *D*), with corresponding benefits and costs as defined in the literature on public goods^58–61^ (while the term public-goods game is usually used for games with more than two players, we here consider a simplified setting with two players). Alternatively, cooperating may mean producing a private good for oneself which happens to produce a by-product benefit for others, where benefits and costs are defined in a different manner^62,63^; as shown in Section 5 of the Supplementary Information, the introduction of heterogeneity in such a private-good model does not qualitatively affect the predictions, which is why it is not considered here.

**Table 1:** Asymmetric bimatrix game representing payoffs of the strong player (*s*) and the weak player (*w*), depending on whether each of the players cooperates (*C*) or defects (*D*).

		Weak player (*w*)	
		Cooperate (*C*)	Defect (*D*)
Strong player (*s*)	Cooperate (*C*)	*b*^*s*^(*C*, *C*) − *c*, *b*^*w*^(*C*, *C*) − *c*	*b*^*s*^(*C*, *D*) − *c*, *b*^*w*^(*C*, *D*)
	Defect (*D*)	*b*^*s*^(*D*, *C*), *b*^*w*^(*D*, *C*) − *c*	*b*^*s*^(*D*, *D*), *b*^*w*^(*D*, *D*)

*b*^*i*^(*x*, *y*) denotes the benefit that player *i* = *s*, *w* obtains from the public good when player *s* adopts strategy *x* = *C*, *D* and player *w* adopts strategy *y* = *C*, *D*. The cooperation costs are denoted by *c*.

Specifically, *b*^*i*^(*x*, *y*) denotes the benefit that player *i* = *s*, *w* obtains from the public good when player *s* adopts strategy *x* = *C*, *D* and player *w* adopts strategy *y* = *C*, *D*. Furthermore in Table 1, *c* denotes cooperation costs, with *c* > 0. Define by ${{\rm{\Delta }}}_{x}^{i}$ (with ${{\rm{\Delta }}}_{x}^{i} > 0$) the added benefit of cooperating rather than defecting (in short: added benefit) for focal player *i* = *s*, *w*, when the other player cooperates (*x* = 0) or defects (*x* = 1), so that ${{\rm{\Delta }}}_{0}^{w}={b}^{w}(D,C)-{b}^{w}(D,D)$, ${{\rm{\Delta }}}_{1}^{w}={b}^{w}(C,C)-{b}^{w}(C,D)$, ${{\rm{\Delta }}}_{0}^{s}={b}^{s}(C,D)-{b}^{s}(D,D)$, and ${{\rm{\Delta }}}_{1}^{s}={b}^{s}(C,C)-{b}^{s}(D,C)$. These added benefits depend on both the degree of adversity (in short: adversity) and on the degree of heterogeneity (in short: heterogeneity), which we now define.

### Adversity

Denoting in general adversity by *a*, in both modelling variants for minimal adversity it is the case that ${{\rm{\Delta }}}_{0}^{i}={{\rm{\Delta }}}_{1}^{i}$ (with *i* = *s*, *w*), meaning that with minimal adversity, a player of a given type contributes the same to the public good, whether or not the other player contributes as well. Furthermore, in both modelling variants the effect of adversity on the added benefits is monotonic; specifically, it is the case that $\partial {{\rm{\Delta }}}_{1}^{i}/\partial a > 0$, and $\partial {{\rm{\Delta }}}_{0}^{i}/\partial a < 0$, so that as adversity is increased, a second cooperating player of a given type contributes more than a first cooperating player (this is with some abuse of notation, as in the defence variant the variable measuring adversity can only take on integer values). Put otherwise, as adversity is increased, a player who defects from joint cooperation becomes to a larger extent the victim of his own defection (referred to as the boomerang effect^16,38^); at the same time a player who deviates from joint defection by cooperating benefits to a smaller extent.

As detailed in the Methods section, in the production variant, adversity is measured by the degree of complementarity between the players’ efforts, which measures how much a second cooperating player in a pair contributes to the public good compared to a first cooperating player (and thus measures to what extent a player’s contribution is pivotal). In the defence variant, the degree of complementarity between the players’ contributions to the public good is instead fixed. Players in a pair face random attacks, and a player contributes to the public good when defending the public good (=cooperating), but also when not defending it (=defecting) but never being attacked. Adversity is now measured by the number of random attacks facing the players. For a large number of attacks, defecting means not contributing to the public good with probability approaching 1, and the extent to which a second cooperating player contributes to the public good compared to the first cooperating player is reflected by the fixed degree of complementarity. For a lower number of random attacks, a second cooperating player contributes less to the public good, because a defecting player is targeted with a probability lower than 1. The number of attacks therefore has a similar effect as the variable degree of complementarity in the production variant, in making each player’s effort more pivotal.

### Heterogeneity

The strong and the weak player differ in the sense that the strong player gains more from cooperating than defecting than the weak player, whatever the action taken by the other player; put otherwise, for *x* = 0, 1, it is the case that ${{\rm{\Delta }}}_{x}^{s}\ge {{\rm{\Delta }}}_{x}^{w}$. As explained in detail in the Methods section, for both modelling variants we separately consider the case where the strong player contributes more to the public good than the weak player, where the strong player obtains a larger share of the public good than the weak player, and where the strong player has lower cooperation costs than the weak player (where the latter case is analytically equivalent to a case with heterogeneous benefits and homogeneous costs, and therefore can still be analysed within this framework of Table 1). In each of these cases, heterogeneity is caught by a single parameter *h*, where for vanishing heterogeneity we have ${{\rm{\Delta }}}_{x}^{s}={{\rm{\Delta }}}_{x}^{w}$ for *x* = 0, 1, and where it is the case that $\frac{\partial {{\rm{\Delta }}}_{x}^{s}}{\partial h} > 0$ and $\frac{{{\rm{\Delta }}}_{x}^{s}}{\partial h} < 0$, such that all else equal larger heterogeneity increases (decreases) how much the strong (weak) player gains from cooperating rather than defecting.

## Results

### Strict equilibria

The question we seek to answer is: what actions do evolutionarily stable strategies (ESSs^64^) prescribe for players in subgames as Table 1, when players face a particular level of cooperation costs, a particular degree of adversity, and a particular degree of heterogeneity. As shown in the Methods section, to answer this question, it suffices to look for strict equilibria of the subgames. It is straightforward to see that three strict equilibria are possible, as summarized in Table 2. Given that ${{\rm{\Delta }}}_{1}^{s}\ge {{\rm{\Delta }}}_{0}^{s}$ and that ${{\rm{\Delta }}}_{0}^{s}\ge {{\rm{\Delta }}}_{0}^{w}$, it follows that ${{\rm{\Delta }}}_{1}^{s}\ge {{\rm{\Delta }}}_{0}^{w}$. As a strict equilibrium where the weak player cooperates and the strong player defects would require that ${{\rm{\Delta }}}_{1}^{s} < c$ and ${{\rm{\Delta }}}_{0}^{w} > c$, such an equilibrium is impossible. The remaining possible strict equilibria are therefore those where both players cooperate (joint cooperation), where both players defect (joint defection), and where the strong player cooperates and the weak player defects. Joint cooperation is a strict equilibrium when ${{\rm{\Delta }}}_{1}^{s} > c$ and ${{\rm{\Delta }}}_{1}^{w} > c$; yet, as ${{\rm{\Delta }}}_{1}^{s} > {{\rm{\Delta }}}_{1}^{w}$, the condition for such an equilibrium can be expressed as ${{\rm{\Delta }}}_{1}^{w} > c$. Joint defection is a strict equilibrium when ${{\rm{\Delta }}}_{0}^{s} < c$ and ${{\rm{\Delta }}}_{0}^{w} < c$; yet, as ${{\rm{\Delta }}}_{0}^{s} > {{\rm{\Delta }}}_{0}^{w}$, the condition for such an equilibrium can be expressed as ${{\rm{\Delta }}}_{0}^{s} < c$. A strict equilibrium where the strong player cooperates and the weak player defects exists when ${{\rm{\Delta }}}_{0}^{s} > c$ and ${{\rm{\Delta }}}_{1}^{w} < c$, which is only possible when ${{\rm{\Delta }}}_{0}^{s} > {{\rm{\Delta }}}_{1}^{w}$.

**Table 2:** Depending on the relation between cooperation costs *c* on the one hand, and the added benefit of cooperating rather than defecting to the strong player when the other player defects (${{\rm{\Delta }}}_{0}^{s}$) and the added benefit of cooperating rather than defecting to the weak player when the other player cooperates (${{\rm{\Delta }}}_{1}^{w}$): strict equilibria obtained, and corresponding form of the game in Table 1.

Relation added benefits to cooperation costs *c*	Strict equilibria	Game form
${{\rm{\Delta }}}_{0}^{s} > c$, ${{\rm{\Delta }}}_{1}^{w} > c$	Both players cooperate	Prisoner’s Dilemma
${{\rm{\Delta }}}_{0}^{s} < c$, ${{\rm{\Delta }}}_{1}^{w} < c$	Both players defect	Harmony Game
${{\rm{\Delta }}}_{0}^{s} < c < {{\rm{\Delta }}}_{1}^{w}$	Equilibrium 1: Both players cooperate; Equilibrium 2: Both players defect	Stag Hunt
${{\rm{\Delta }}}_{1}^{w} < c < {{\rm{\Delta }}}_{0}^{s}$	Strong player cooperates, weak player defects	Free-Rider game

### Types of games played

From the previous it follows that the game in Table 1, following standard taxonomies of games^58–60,65^, can take on four different forms. When ${{\rm{\Delta }}}_{0}^{s} > c$ and ${{\rm{\Delta }}}_{1}^{w} > c$, the only strict equilibrium is joint cooperation, and we have so-called Harmony Game^66^. When ${{\rm{\Delta }}}_{0}^{s} < c$ and ${{\rm{\Delta }}}_{1}^{w} < c$, the only strict equilibrium is joint defection, and we have a Prisoner’s Dilemma^67^. When ${{\rm{\Delta }}}_{0}^{s} < {{\rm{\Delta }}}_{1}^{w}$ and ${{\rm{\Delta }}}_{0}^{s} < c < {{\rm{\Delta }}}_{1}^{w}$, both joint cooperation and joint defection are equilibria, and we have a Stag Hunt^68^. Heterogeneity means that the strong player benefits more from contributing to the public good than the weak player, adversity means that a second cooperating player in a pair contributes more than a first cooperating player; the necessary condition ${{\rm{\Delta }}}_{0}^{s} < {{\rm{\Delta }}}_{1}^{w}$ for a Stag Hunt thus requires that adversity has more impact than heterogeneity, as a weak second cooperating player then benefits more than a strong first cooperating player. When ${{\rm{\Delta }}}_{1}^{w} < {{\rm{\Delta }}}_{0}^{s}$ and ${{\rm{\Delta }}}_{1}^{w} < c < {{\rm{\Delta }}}_{0}^{s}$ the game only has a strict equilibrium where the strong player cooperates and the weak player defects, and we refer to it as a Free-Rider game (referred to as exploitation of the great by the small in the classical analysis of collective action problems by Olson^69^). A necessary condition for a Free-Rider game is that heterogeneity is sufficiently strong compared to adversity, such that a second weak cooperating player benefits less than a first strong cooperating player.

### Comparison of vanishing and large heterogeneity

**Figure 1:**
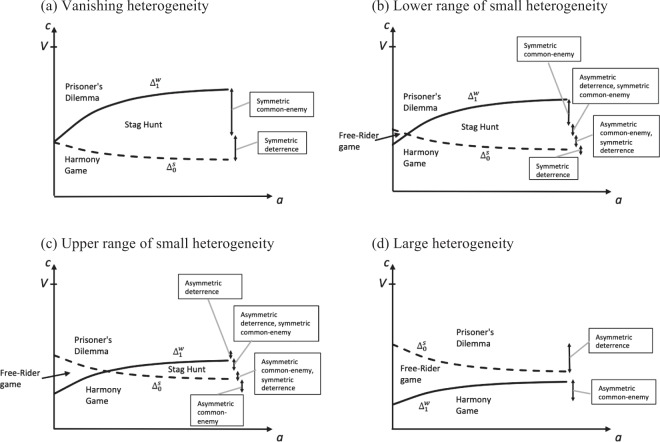
For the game in Table 1, as a function of adversity *a*, added benefit of cooperating rather than defecting for the weak player if the other player cooperates (${{\rm{\Delta }}}_{1}^{w}$), and for the strong player if the other player defects (${{\rm{\Delta }}}_{0}^{s}$). Depending on the relation between cooperation costs and these added benefits, the form of the game played is indicated (see Table 2). Depending on how adversity affects the form of the game played, it is indicated whether as a function of the cooperation costs the (**a**) symmetric common-enemy or (**a**) symmetric deterrence effect applies, as defined in Table 3. The particular case represented is the defence variant of the model (so that adversity *a* is measured by the number of random attacks *A*, which in the graphs range from 1 to 7 attacks) with heterogeneous shares obtained from the public good (see Methods section), where *V* = 1 and $k=3/4$. Figures (**a**) to (**d**) represent cases with increasingly high heterogeneity ((**a)** *h* = 0.5; (**b**) *h* = 0.68; (**c**) *h* = 0.78; (**d**) *h* = 0.99).

As a starting point to assess the effect of higher adversity (where the possible types of effects are summarised in Table 3), and how this effect is impacted by heterogeneity, we compare vanishing and large heterogeneity. Figure 1 considers as an example the defence variant of the model, with heterogeneity in the form of different shares of the public good obtained (for details, see the Methods section), and represents ${{\rm{\Delta }}}_{0}^{s}$ and ${{\rm{\Delta }}}_{1}^{w}$ as a function adversity in the form of the number of random attacks *A* (where for ease of representation, we present ${{\rm{\Delta }}}_{0}^{s}$ and ${{\rm{\Delta }}}_{1}^{w}$ as continuous functions, even though *A* can only take on integer values). Figure 1(a) represents the limit case where heterogeneity is vanishingly small, as analysed elsewhere^52^. In this case, for minimal adversity we have ${{\rm{\Delta }}}_{1}^{w}={{\rm{\Delta }}}_{0}^{s}$. Representing cooperation costs along the Y-axis and comparing them to ${{\rm{\Delta }}}_{0}^{s}$ and ${{\rm{\Delta }}}_{1}^{w}$, one can now indicate in Fig. 1(a) whether the game is a Prisoner’s Dilemma, a Stag Hunt, or a Harmony Game. Starting from low adversity, when cooperation costs are large no cooperation takes place (Prisoner’s Dilemma). With higher adversity, a first cooperating player benefits even less from unilaterally cooperating ($\partial {{\rm{\Delta }}}_{0}^{s}/\partial a < 0$), but a second cooperating player has more and more to lose from unilaterally deviating from joint cooperation ($\partial {{\rm{\Delta }}}_{1}^{w}/\partial a > 0$). Because of this a common-enemy effect is obtained, as joint cooperation now also becomes a strict equilibrium (Stag Hunt). We refer to this as the *symmetric common-enemy effect*. Starting again from low adversity, when cooperation costs are instead small, joint cooperation takes place (Harmony Game). With higher adversity, a second cooperating player loses even more when failing to cooperate ($\partial {{\rm{\Delta }}}_{1}^{w}/\partial a > 0$) and joint cooperation continues to be a strict equilibrium, but a first cooperating player has less to gain from unilaterally cooperating ($\partial {{\rm{\Delta }}}_{0}^{s}/\partial a < 0$), so that joint defection also becomes a strict equilibrium (Stag Hunt). We refer to this as the *symmetric deterrence effect*.

**Table 3:** Possible cases for the effect of higher adversity on the probability of cooperation identified across all modelling variants considered in the Methods section; definition of these effects in terms of changes in the form of the game (see Table 2).

	Effect of higher adversity	Description in terms of change in game form
Case 1	Symmetric common-enemy effect	From Prisoner’s Dilemma to Stag Hunt
Case 2	First asymmetric deterrence, then symmetric common-enemy effect	From Free-Rider game to Prisoner’s Dilemma, and finally Stag Hunt
Case 3	First asymmetric common enemy, then symmetric deterrence effect	From Free-Rider game to Harmony Game, and finally Stag Hunt
Case 4	Symmetric deterrence effect	From Harmony Game to Stag Hunt
Case 5	Asymmetric deterrence effect	From Free-Rider game to Prisoner’s Dilemma
Case 6	Asymmetric common-enemy effect	From Free-Rider game to Harmony Game

Starting from Fig. 1(a), larger heterogeneity shifts ${{\rm{\Delta }}}_{0}^{s}$ upwards and ${{\rm{\Delta }}}_{1}^{w}$ downwards. Figure 1(d) represents the case where heterogeneity is large, and where shifting has taken place to such an extent that it is the case that ${{\rm{\Delta }}}_{0}^{s} > {{\rm{\Delta }}}_{1}^{w}$ for any degree of adversity. Start from small adversity and consider cooperation cost ranges ${{\rm{\Delta }}}_{1}^{w} < c < {{\rm{\Delta }}}_{0}^{s}$, such that the game is a Free-Rider game. Consider first large cooperation costs. As we increase adversity, it now continues to be the case that a weak second cooperating player is better off defecting (${{\rm{\Delta }}}_{1}^{w} < c$), but it also becomes the case that a strong first cooperating player is also better off defecting (${{\rm{\Delta }}}_{0}^{s} < c$), so that the game becomes a Prisoner’s Dilemma; we refer to this as the *asymmetric deterrence effect*. Next, consider small cooperation costs. For higher adversity, it is still the case that a strong first cooperating player is better off cooperating $(c < {{\rm{\Delta }}}_{0}^{s})$, but a weak second cooperating player also becomes better off cooperating $(c < {{\rm{\Delta }}}_{1}^{w})$, so that the game becomes a Harmony Game; we refer to this as the *asymmetric common-enemy effect* (this distinction between symmetric and asymmetric effects is similar to the effect of the degree of relatedness on the probability of cooperation in the treatment of Hamilton’s rule^70^). As comparison of Fig. 1(a,d) shows, compared to the case of vanishing heterogeneity, large heterogeneity completely reverses the incidence of the common-enemy and deterrence effects as a function of the cooperation costs.

### Non-monotonic effects of adversity for small heterogeneity

Figure 1(b,c) represent scenarios where heterogeneity is not vanishing, but at the same time not as large as in Fig. 1(d), where we refer to these scenarios as small heterogeneity. In such scenarios, higher adversity can have a non-monotonic effect on the probability of cooperation. Figure 1(b) represents a scenario within the lower range of small heterogeneity. In this case, just as the case in Fig. 1(a) (vanishing heterogeneity), for the upper ranges of high cooperation costs, the symmetric common-enemy effect applies, and for the lower ranges of small cooperation costs the symmetric deterrence effect applies. Yet, for the lower ranges of large cooperation costs, as adversity is increased, first the asymmetric deterrence effect applies, and only then the symmetric common-enemy effect. For the upper ranges of low cooperation costs, as adversity is increased, first the asymmetric common-enemy effect applies, and only then the symmetric deterrence effect. Figure 1(c) represents a scenario within the upper range of smaller heterogeneity. In this case, just as in Fig. 1(d) (large heterogeneity), for the upper ranges of high cooperation costs, the asymmetric deterrence effect applies, and for the lower ranges of small cooperation costs the asymmetric common-enemy effect applies. Yet, for intermediate cost levels, the same non-monotonic effects as in Fig. 1(b) are obtained.

### Intuition for non-monotonic effects

While Fig. 1(a,d) show that the incidence of the common-enemy and deterrence effect is reversed for large heterogeneity, the non-monotonic effects of adversity for small heterogeneity in Fig. 1(b,c) complicate the results. Yet, we now argue that the non-monotonic effects are part of the same mechanism where sufficiently large heterogeneity reverses the incidence of the common-enemy and deterrence effects. Indeed, as higher adversity make each player’s contribution to the public good more pivotal, a weak second cooperating player may then still contribute more than a strong first cooperating player, thus undoing the effect of heterogeneity, explaining why in Fig. 1(b,c) the symmetric effects apply for high adversity. In contrast, low adversity means that heterogeneity will have a relatively large impact, and that the asymmetric effects are obtained (with the corresponding reversed incidence of the common-enemy and deterrence effects), even though heterogeneity may be small in absolute terms. The incidence of the common-enemy and deterrence effects therefore depends on how large the degree of heterogeneity is *relative* to the degree of adversity. In Fig. 1(d), heterogeneity is so large that high adversity can never undo it, and the asymmetric effects apply. In Fig. 1(b,c), heterogeneity is relatively large when adversity is low (asymmetric effects), but heterogeneity becomes relative small when adversity is high (symmetric effects).

### Detailed results

Figure 1 represents the results for the defence variant, when heterogeneity takes the form that players obtain different shares from the public good. While the results of all modelling variants have common features, the precise details differ, as represented in Table 4. Common features are that as heterogeneity is increased, the asymmetric common-enemy and deterrence effects apply for a wider range of degrees of adversity; this follows naturally from the fact that heterogeneity shifts ${{\rm{\Delta }}}_{0}^{s}$ upwards, and ${{\rm{\Delta }}}_{1}^{w}$ downwards, as can be seen by comparing Fig. 1(b,c). The way in which the detailed results differ across the variants, is that in some variants for sufficiently large heterogeneity, the asymmetric common-enemy and deterrence effects become the only possible effects (as is the case in Fig. 1(d)), whereas in other variants even for a maximal heterogeneity, the symmetric effects continue to apply as long as adversity is sufficiently high.

**Table 4:** Summary of the results for the effect of higher adversity on the probability of cooperation.

		Symmetric common-enemy	Asymmetric deterrence	Asymmetric deterrence	Asymmetric deterrence
		Asymmetric deterrence, then symmetric common-enemy	Asymmetric deterrence, then symmetric common-enemy	Asymmetric deterrence, then symmetric common-enemy	
		Asymmetric common-enemy, then symmetric deterrence	Asymmetric common-enemy, then symmetric deterrence	Asymmetric common-enemy, then symmetric deterrence	
		Symmetric deterrence	Symmetric deterrence	Asymmetric common-enemy	Asymmetric common-enemy
Model Variant	Type of Heterogeneity				
Production	Capability	X			
	Shares	X	X		
	Cooperation costs	X	X		
Defence	Capability	X			
	Shares	X	X	X	X
	Cooperation costs	X	X	X	X


The rows represent the different modelling variants in the Methodology section. Each column represents which of the effects of a higher adversity listed in Table 3 occurs, where these effects are ordered according to their incidence as a function of the cooperation costs (from large to small cooperation costs). The columns are ordered representing larger heterogeneity from left to right. Specifically for the defence variant of the model, the case with a large fixed degree of complementarity is represented.

These differences in results are explained as follows. First, the results differ between the production and defence variants for the following reason. In the production variant, where adversity is measured by the degree of complementarity, complementarity can become so large that a first cooperating player contributes vanishingly little compared to a second cooperating player; in this case, the effect of heterogeneity is undone, explaining why in the production variant symmetric effects continue to be obtained for high adversity. In the defence variant on the contrary, the fixed degree of complementarity between the players’ contributions need not be maximal. For this reason, even with maximal adversity (=large number of random attacks), the extent to which players’ contributions are pivotal is still not maximal. The consequence is that even high adversity does not undo the effect of heterogeneity, explaining why for large heterogeneity (cf. Fig. 1(d)) only the asymmetric effects may apply.

Second, heterogeneity modelled as a difference in capability of contributing to the public good leads to different effects than the other two types of heterogeneity considered. Even if players differ considerably in their capabilities to contribute to the public good, when a second cooperating player’s effort becomes to a large extent critical, this undoes the effect of heterogeneity and the symmetric effects are obtained. However, when heterogeneity takes the form of players obtaining different shares of the public good or incurring different cooperation costs, heterogeneity can always be so large that a first cooperating player who is strong is more inclined to contribute to the public good than a second cooperating player who is weak, whatever the degree of adversity (cf. Fig. 1(d)). This explains why in these cases, for large heterogeneity, only the asymmetric effects may apply.

## Discussion

The game-theoretic model in this report allows not only for a common-enemy effect, but also for a deterrence effect of a harsher environment. Moreover, how the incidence of these two effects of a harsher environment changes as a function of the cooperation costs, depends on the degree to which the players are heterogeneous: if heterogeneity is relatively small, the common-enemy effect applies for large cooperation costs, and the deterrence effect applies for small cooperation costs; if heterogeneity is instead relatively large, it is the deterrence effect that applies for large cooperation costs, and the common-enemy effect for small cooperation costs. The model thus provides detailed predictions for how the phenotypic plasticity of cooperative behaviour may be influenced by the harshness of the environment, the cooperation costs, and the degree of heterogeneity between the players.

It should be stressed that the model for which these predictions are obtained is extremely stylised, with several simplifying assumptions. Costs of plasticity^71^, such as information-acquisition costs to determine the cooperation costs, the degree of adversity and the degree heterogeneity, were not considered (though experiments suggest that even simple organisms are able to overcome these costs^17^). In the model, players cooperate in pairs, whereas in reality cooperating groups may have any size (an extension in the Supplementary Information (Section 6) suggests that the results extend to larger groups, even though these give rise to additional non-monotonic effects of adversity). Additionally, the formation of groups may itself be subject to evolution, rather than groups being given as in the model^72^. What players produce in the model is a pure public good; in reality, players who do not cooperate may obtain fewer benefits than players who defect (e.g., free-riding on the collective hunt of other predators in one’s group may lead one to obtain a smaller share of the prey), and congestion may take place as the number of cooperators in a group increases (e.g., when more predators participate in a collective hunt, the prey needs to be shared among more predators)^73^. Finally, while in the model investment in the public good is an all-or-nothing one-dimensional decision, in reality cooperators’ efforts may be continuous^58^. Though the expectation is that the stylised model in this report reveals mechanisms that are at work in much more complex reality, relaxing the mentioned simplifying assumptions deserves separate attention. Most of all, while in the model the focus is on the effect of harsher environments on the probability of cooperation, future research needs to investigate how this effect interacts with standard rationales for the evolution of cooperation, including crucially network reciprocity. Moreover, an avenue for future research is to explore the effect of harsher environments when players are able to collaborate, in being able to make coordinated moves towards strategies that are mutually beneficial^74,75^.

We end by exploring how the results of the model could be translated into testable hypotheses. While adversity may be quantified by predation risk in the defence variant, or by the size of a prey in the production variant (where a larger prey makes each predator’s contribution to a collective hunt more pivotal)^16^, and while heterogeneity may also have a direct biological interpretation, cooperation costs may be difficult to quantify (a similar problem arises when deriving hypotheses from theories on the formation of coalitions and alliances^76^). The level of the cooperation costs is moreover only defined relative to the benefits obtained from the public good, which also do not have a direct biological interpretation. Moreover, even if an appropriate proxy variable could be found to account for relative cooperation costs, such a variable need not relate in a linear way to fitness, and therefore to the payoffs in Table 1. For this reason, a large size of any such proxy variable need not translate into what corresponds to large cooperation costs in the model, so that it may not be possible to observe all the cases in the model by using a proxy variable for cooperation costs. Also, even if one can find a proxy variable that is not subject to this problem, one additionally needs to ensure that the different variables are not correlated. For instance, a larger prey may not only make each predator’s contribution to a cooperative hunt more pivotal and may thus not only measure adversity, but may also increase cooperation costs at the same time. We conclude that turning the predictions of our model into testable hypotheses requires separate attention.

## Methods

Two modelling variants of the effect of a harsher environment are considered, namely a production variant and a defence variant^52^. For each of these models, three types of heterogeneity are considered, namely heterogeneity in the capability of contributing to the public good, heterogeneity in the share of the public good obtained, and heterogeneity in cooperation costs. As reported in the Results section, qualitatively similar results are obtained for each of the cases. Formal descriptions of the results, and all the proofs, are contained in the Supplementary Information (Sections 3 and 4).

### Evolutionary game

We consider an infinitely large population that reproduces asexually. In each period, with a given probability, the player is assigned the role of a strong player or of a weak player. Players are assigned to a role fully independently of the roles they may have had in the past, and the fraction of strong and weak players in the population is not subject to evolution. The population is repeatedly and randomly matched in pairs, where pairs are formed independently of the roles of the players. A strategy of a player tells the player whether to cooperate or defect in each possible subgame that may be formed, depending on the player’s own type and the type of the player to whom he is matched, on the degree of adversity, and on the degree of heterogeneity. The change in the fraction of players adopting a strategy is assumed to follow the continuous replicator dynamics^77^.

### Evolutionarily stable strategies and strict equilibria

Any ESS of the specified asymmetric game must be a strict, and therefore a pure-strategy equilibrium^78^. Moreover, in order to have a strict equilibrium of the entire game, players’ strategies in each of the subgames that can be formed must be strict mutual best responses, so that to any ESS must correspond a strict equilibrium in each possible subgame^79^. Since our interest is in the effect of heterogeneity, Table 1 considers a subgame where an asymmetric pair is formed. The cases where a symmetric pair is formed is already analysed in the symmetric version of the model in this report^52^.

### Production variant

The production variant may be seen as a stylised representation of cooperative hunting^53,54^, where the good produced is the prey caught. In the version of this variant with vanishing heterogeneity, if both players in a pair cooperate, they both obtain payoff *V* from the public good (meaning *b*^*s*^(*C*, *C*) = *b*^*w*^(*C*, *C*) = *V*); if both players defect they both obtain payoff 0 (meaning *b*^*s*^(*D*, *D*) = *b*^*w*^(*D*, *D*) = 0). If one player cooperates and the other one defects, both players obtain value (1 − *k*)*V* from the public good (meaning *b*^*s*^(*C*, *D*) = *b*^*w*^(*C*, *D*) = *b*^*s*^(*D*, *C*) = *b*^*w*^(*D*, *C*) = (1 − *k*)*V*). It is the case that $1/2\le k\le 1$, where *k* is the *degree of complementarity* between the players’ efforts^80^, and reflects the extent to which a second cooperating player in a pair is pivotal compared to a first cooperating player. With *k* = 1/2, pivotality is minimal, and a first and second cooperating player contribute the same (in this case, the game is a two-player version of the linear public-goods game^58^, with benefit of the public good equal to *πrc*/2, where *π* is the number of cooperating players in the pair and *r* is the multiplication factor; our model is obtained by assuming that *rc* = *V*). With *k* = 1 pivotality is maximal, and a second cooperating player contributes the entire value of the public good.

#### Heterogeneous capability to contribute to the public good

It continues to be the case that *b*^*s*^(*C*, *C*) = *b*^*w*^(*C*, *C*) = *V* and that *b*^*s*^(*D*, *D*) = *b*^*w*^(*D*, *D*) = 0. Yet, it is now the case that *b*^*s*^(*C*, *D*) = *b*^*w*^(*C*, *D*) = 2(1 − *k*)*hV*and that *b*^*s*^(*D*, *C*) = *b*^*w*^(*D*, *C*) = 2(1 − *k*)(1 − *h*)*V*, with $1/2\le h\le 1$, where *h* measures the degree of heterogeneity. Specifically, *h* reflects the extent to which the players have heterogeneous capabilities of contributing to the public good, where the larger *h*, the more a strong player that cooperates alone contributes to the public good compared to a weak player that cooperates alone. When *h* = 1/2, heterogeneity vanishes; when *h* = 1, heterogeneity is maximal and a weak player who cooperates alone does not contribute anything.

#### Heterogeneous shares obtained from the public good

In this case, players have the same capability of contributing to the public good, and heterogeneity *h*, with $1/2\le h\le 1$, reflects the share of the total value of the public good that is obtained by the strong player. In particular, we have *b*^*s*^(*C*, *C*) = *h*2*V*, *b*^*s*^(*C*, *D*) = *b*^*s*^(*D*, *C*) = *h*2(1 − *k*)*V*, *b*^*s*^(*D*, *D*) = 0; also, *b*^*w*^(*C*, *C*) = (1 − *h*)2*V*, *b*^*w*^(*C*, *D*) = *b*^*w*^(*D*, *C*) = (1 − *h*)2(1 − *k*)*V*, *b*^*w*^(*D*, *D*) = 0. When *h* = 1/2, the case with vanishing heterogeneity is again obtained. When *h* = 1, heterogeneity is maximal, and e.g. when both players cooperate, of the total value that is produced for the players in the absence of heterogeneity (i.e. *V* for each player, or 2*V* in total), the strong player obtains 2*V* and the weak player obtains nothing.

#### Heterogeneous cooperation costs

In this case, benefits are the same as with vanishing heterogeneity, but the strong and the weak player have different cooperation costs. In particular, we assume that a strong player incurs costs *c*^*s*^ = 2(1 − *h*)*c*, whereas a weak player incurs costs *c*^*w*^ = 2*hc*, with $1/2\le h\le 1$. In this manner, average cooperation costs within a heterogeneous group equal $1/2(2hc)+1/2(2(1-h)c)=c$. As shown in the Supplementary Information, as only the level of the cooperation costs relative to benefits matters for our results, this case is analytically equivalent to a case where players have the same average cooperation costs, but have heterogeneous benefits.

### Defence variant

In the defence variant, the number of players that contributes to the public good relates to the value obtained from the public good in exactly the same way as in the production model, where the degree of complementarity is now kept fixed. Yet, cooperating or defecting now relates in a stochastic way to contributing or not contributing to the public good. In particular, the defence variant represents in a stylised way a situation of circular defence^81^. Each player in a pair is positioned on one of the two sides of a common territory. Cooperating (*C*) means doing effort to defend one’s side, defecting (*D*) means not doing any effort. The players face a number of random attacks *A* (with *A* ≥ 1), taking the form of sampling with replacement of the two sides of the common territory.

With probability 1/2^*A*^, the individual player is never attacked, and with probability [1 − 1/2^*A*^], this player is attacked at least once. The individual player contributes to the public good when defending, but also when not defending and never being attacked. With vanishing heterogeneity, it is the case that *b*^*s*^(*C*, *C*) = *b*^*w*^(*C*, *C*) = *V*. Furthermore, ${b}^{s}(D,D)={b}^{w}(D,D)=\frac{2}{{2}^{A}}V(1-k)$ (where $\frac{2}{{2}^{A}}$ is the probability that only one player is attacked, so that the other player still contributes). Finally, *b*^*s*^(*C*, *D*) = *b*^*w*^(*C*, *D*) = *b*^*s*^(*D*, *C*) = *b*^*w*^(*D*, *C*) = $\frac{1}{{2}^{A}}V+[1-\frac{1}{{2}^{A}}](1-\,k)V$, where $\frac{1}{{2}^{A}}$ is the probability that a non-defending player in a pair is never attacked and still contributes. The number of random attacks measures adversity in the sense that just like the variable degree of complementarity in the production variant, it makes each player’s effort more pivotal, in that for a large number of random attacks a second cooperating player adds more value to the public good compared to a first cooperating player. The same three forms of heterogeneity are now considered as for the production model.

#### Heterogeneous capability to contribute to the public good

In this case, we have *b*^*s*^(*C*, *C*) = *b*^*w*^(*C*, *C*) = *V* and *b*^*s*^(*D*, *D*) = *b*^*w*^(*D*, *D*) $=\frac{1}{{2}^{A}}2(1-k)hV+\frac{1}{{2}^{A}}2(1-k)(1-h)V\,=\frac{2}{{2}^{A}}(1-k)V$. When the strong player cooperates and the weak player defects, ${b}^{s}(C,D)={b}^{w}(C,D)=\frac{1}{{2}^{A}}V+[1-\frac{1}{{2}^{A}}]2(1-\,k)hV$. When the weak player cooperates and the strong player defects, ${b}^{s}(D,C)={b}^{w}(D,C)=\frac{1}{{2}^{A}}V+[1-\frac{1}{{2}^{A}}]2(1-\,k)(1-h)V$.

#### Heterogeneous shares obtained from the public good

Here, *b*^*S*^(*C*, *C*) = *h*2*V*, *b*^*w*^(*C*, *C*) = (1 − *h*)2*V*, ${b}^{s}(D,D)=h2\frac{2}{{2}^{A}}\times$ $(1-k)V$, and ${b}^{w}(D,D)=(1-h)2\frac{2}{{2}^{A}}(1-k)V$. Also, ${b}^{s}(C,D)={b}^{s}(D,C)=\frac{1}{{2}^{A}}h2V+[1-\frac{1}{{2}^{A}}]h2(1-\,k)V$, and ${b}^{w}(C,D)={b}^{w}(D,C)=\frac{1}{{2}^{A}}(1-h)2V+[1-\frac{1}{{2}^{A}}](1-h)2(1-\,k)V$.

#### Heterogeneous cooperation costs

In this case benefits are the same as in the defence variant with vanishing heterogeneity, and heterogeneous cooperation costs are constructed in the same way as for the production model with heterogeneous cooperation costs.

## Supplementary information

Supplementary information

## References

[1] Dugatkin, L. A. *Cooperation Among Animals: an Evolutionary Perspective*. (Oxford University Press, 1997).

[2] Dugatkin LA (2002). Animal cooperation among unrelated individuals. Naturwiss..

[3] Sachs JL, Mueller UG, Wilcox TP, Bull JJ (2004). The evolution of cooperation. Q. Rev. Biol..

[4] Lehmann L, Keller L (2006). The evolution of cooperation and altruism – a general framework and a classification of models. J. Evol. Biol..

[5] Nowak MA (2006). Five rules for the evolution of cooperation. Science.

[6] Perc M (2017). Statistical physics of human cooperation. Phys. Rep..

[7] Capraro V, Perc M (2018). Grand challenges in social physics: in pursuit of moral behavior. Front. Phys..

[8] Trivers RL (1971). The evolution of reciprocal altruism. Q. Rev. Biol..

[9] Nowak MA, Sigmund K (2005). Evolution of indirect reciprocity. Nature.

[10] Hamilton WD (1964). The genetical evolution of social behavior I. J. Theor. Biol..

[11] Traulsen A, Nowak MA (2006). Evolution of cooperation by multilevel selection. PNAS.

[12] Nowak MA, May RM (1992). Evolutionary games and spatial chaos. Nature.

[13] West Eberhard MJ (1975). The evolution of social behavior by kin selection. Q. Rev. Biol..

[14] Brown, J. L. Cooperation: a biologist’s dilemma in *Advanced in the Study of Behavior* (ed. Rosenblatt, J. S.) 1–37 (Academic Press, 1983).

[15] Kropotkin, P. *Mutual Aid*. (Heinemann, 1902).

[16] Mesterton-Gibbons M, Dugatkin LA (1992). Cooperation among unrelated individuals: evolutionary factors. Q. Rev. Biol..

[17] Kümmerli R, Jiricny N, Clarke LS, West SA, Griffin AS (2009). Phenotypic plasticity of a cooperative behaviour in bacteria. J. Evol. Biol.

[18] Gravilets S (2015). Collective action problem in heterogeneous groups. Phil. Trans. R. Soc. B..

[19] Gavaldà-Miralles A (2014). Impact of heterogeneity and socioeconomic factors on individual behavior in decentralized sharing ecosystems. PNAS.

[20] Szolnoki A, Perc M (2016). Biodiversity in models of cyclic dominance is preserved by heterogeneity in site-specific invasion rates. Sci. Rep..

[21] Kaveh K, McAvoy A, Nowak MA (2019). Environmental fitness heterogeneity in the Moran process. R. Soc. Open Sci..

[22] Lotem A, Fishman MA, Stone L (1999). Evolution of cooperation between individuals. Nature.

[23] Sherratt TN, Roberts G (2001). The importance of phenotypic defectors in stabilizing reciprocal altruism. Behav Ecol..

[24] Fishman MA, Lotem A, Stone L (2001). Heterogeneity stabilizes reciprocal altruism interactions. J. Theor. Biol..

[25] Perc M, Szolnoki A (2008). Social diversity and promotion of cooperation in the spatial prisoner’s dilemma game. Phys. Rev. E.

[26] Szolnoki A, Perc M, Szabó G (2008). Diversity of reproduction rate supports cooperation in the prisoner’s dilemma game on complex networks. Eur. Phys. J. B.

[27] Perc M (2011). Success-driven distribution of public goods promotes cooperation but preserves defection. Phys. Rev. E.

[28] Perc M (2011). Does strong heterogeneity promote cooperation by group interactions?. New J. Phys..

[29] Qin J, Chen Y, Fu W, Kang Y, Perc M (2018). Neighborhood diversity promotes cooperation in social dilemmas. IEEE Access.

[30] Rodrigues AMM, Gardner A (2013). Evolution of helping and harming in heterogeneous groups. Evolution.

[31] McAvoy, A. & Hauert, C. Asymmetric evolutionary games. *PLoS Comput. Biol*. **11**(**8**) (2015). 10.1371/journal.pcbi.1004349 PMC4550251 26308326

[32] Maynard Smith J, Price GR (1976). The logic of asymmetric contests. Anim. Behav..

[33] Simmel, G. *Conflict*. (Free Press, 1908).

[34] Coser, L. A. *The Functions of Social Conflict*. (Free Press, 1956).

[35] Heider, F. *The Psychology of Interpersonal Relations*. (John Wiley & Sons, 1958).

[36] Muller EN, Opp K-D (1986). Rational choice and rebellious collective action. Am. Pol. Science Rev..

[37] Bornstein G, Gneezy U, Nagel R (2002). The effect of intergroup competition on group coordination: an experimental study. Games Econ. Behav..

[38] Mesterton-Gibbons M, Dugatkin LA (1997). Cooperation and the prisoner’s dilemma: towards testable models of mutualism versus reciprocity. Anim. Behav..

[39] Strassman JE, Zhu Y, Queller DC (2000). Altruism and social cheating in social amoeba. Dictyostelium discoideum. Nature.

[40] Callaway RG (2002). Positive interactions among alpine plants increase with stress. Nature.

[41] Spieler M (2003). Risk of predation affects aggregation size: study with tadpoles of Phrynomantis microps (Anura: Microhylidae). Anim. Behav..

[42] Krams I, Krama T, Berzins A, Rantala MJ (2010). The risk of predation favors cooperation among breeding prey. Commun. Integr. Biol..

[43] Smaldino PE, Schank JC, McElreath R (2013). Increased costs of cooperation helps cooperators in the long run. Am. Nat..

[44] Maharjan R (2013). The form of a trade-off determines the response to competition. Ecol. Lett..

[45] Smaldino PE (2013). Cooperation in harsh environments and the emergence of spatial patterns. Chaos, Soliton. Fract..

[46] Szolnoki A, Antonioni A, Tomassini M, Perc M (2014). Binary birth-death dynamics and the expansion of cooperation by means of self-organized growth. EPL.

[47] Pérez I, Janssen MA (2015). The effect of spatial heterogeneity and mobility on the performance of social-ecological systems. Ecol. Modell..

[48] Szolnoki A, Chen X (2016). Cooperation driven by success-driven group formation. Phys. Rev. E.

[49] Krams, I. *et al*. The increased risk of predation enhances cooperation. *Proc. R. Soc. Lond. B* **277**, 513–518. 10.1098/rspb.2009.1614 PMC2842689 19846454

[50] Bowles S (2009). Did warfare among ancestral hunter-gatherers affect the evolution of human social behaviors?. Science.

[51] Wakeley J, Nowak M (2019). A two-player iterated survival game. Theor. Pop. Biol..

[52] De Jaegher K, Hoyer B (2016). By-product mutualism and the ambiguous effects of harsher environments: a game-theoretic model. J. Theor. Biol..

[53] Scheel D, Packer C (1991). Group hunting behavior of lions: a search for cooperation. Anim Behav..

[54] Stander P (1992). Cooperative hunting in lions: the role of the individual. Behav. Ecol. Sociobiol..

[55] Gese EM (2001). Territorial defense by coyotes (Canis latrans) in Yellowstone National Park, Wyoming: who, how, where, when, and why. Can. J. Zool..

[56] Rubenstein, D. I. & Nuñez, C. N. Sociality and reproductive skew in horses and zebras in *Reproductive Skew in Vertebrates: Proximate and Ultimate Causes* (eds Hager, R. & Jones, C. B.) 196–226 (Cambridge University Press, 2009).

[57] Port M, Kappeler PM, Johnstone RA (2011). Communal defense of territories and the evolution of sociality. Am. Nat..

[58] Doebeli M, Hauert C (2005). Models of cooperation based on the Prisoner’s Dilemma and the Snowdrift game. Ecol Lett..

[59] Archetti M (2011). Economic game theory for mutualism and cooperation. Ecol. Lett..

[60] Archetti M, Scheuring I (2012). Review: game theory of public goods in one-shot dilemmas without assortment. J. Theor. Biol..

[61] Hauert C, Michor F, Nowak MA, Doebeli M (2006). Synergy and discounting in social dilemmas. J. Theor. Biol..

[62] Caraco T, Brown JL (1986). A game between communal breeders: when is food-sharing stable?. J. Theor. Biol..

[63] Mesterton-Gibbons M (1991). An escape from ‘the prisoner’s dilemma’. J. Math. Biol..

[64] Maynard Smith J, Price GR (1973). The logic of animal conflict. Nature.

[65] Connor RC (1995). Altruism among non-relatives: alternatives to the ‘Prisoner’s Dilemma’. TREE.

[66] Martinez M, Pichler A, Sigmund K (1999). The efficiency of adapting aspiration levels. Proc. R. Soc. B..

[67] Tucker, A. A two-person dilemma in *Readings in Games and Information* (ed. Rasmussen, E.) 7–8 (Blackwell, 1950).

[68] Skyrms, B. *The Stag Hunt and Evolution of Social Structure*. (Cambridge University Press, 2004).

[69] Olson, M. *The Logic of Collective Action: Public Goods and the Theory of Groups*. (Harvard University Press, 1965).

[70] van Veelen M, Allen B, Hoffman M, Simon B, Veller C (2017). Hamilton’s rule. J. Theor. Biol..

[71] Auld JR, Agrawal AA, Relyea RA (2010). Re-evaluating the costs and limits of adaptive phenotypic plasticity. Proc. R. Soc. B.

[72] Garcia T, De Monte S (2012). Group formation and the evolution of sociality. Evolution.

[73] De Jaegher K (2019). Harsh environments: multi-player cooperation with excludability and congestion. J. Theor. Biol..

[74] Newton J (2017). Shared intentions: the evolution of collaboration. Games Econ. Behav..

[75] Rusch H (2019). The evolution of collaboration in symmetric 2x2-games with imperfect recognition of types. Games Econ. Behav..

[76] Bissonnette A (2015). Coalitions in theory and reality: a review of pertinent variables and processes. Behaviour.

[77] Hofbauer, J. & Sigmund, K. *Evolutionary Games and Population Dynamics*. (Cambridge University Press, 1998).

[78] Selten Reinhard (1980). A note on evolutionarily stable strategies in asymmetric animal conflicts. Journal of Theoretical Biology.

[79] Hammerstein P (1981). The role of asymmetries in animal contests. Anim. Behav..

[80] Ray D, Baland J-M, Dagnelie O (2007). Inequality and inefficiency in joint projects. Econ. J..

[81] Jolivet, P., Vasconcellos-Neto, J. & Weinstein, P. Cycloalexy: a new concept in the larval defense of insects. *Insect. Mundi* **4**, 133–141.

