# Supplementary material for: Adversity and cooperation in heterogeneous pairs
Source: Sci Rep. 2019 Jul 15;9:10164. doi: 10.1038/s41598-019-46624-8 (PMC6629845; doi:10.1038/s41598-019-46624-8)
Supplement: Supplementary file 1 — Supplementary information [file 41598_2019_46624_MOESM1_ESM.docx]

Adversity and cooperation in heterogeneous pairs, Kris De Jaegher: Supporting information

This Supporting Information contains the following parts. Section 1 contains a list of main symbols used in the report, and in Sections 2 to 4. Section 2 provides a general framework for describing the results for the defence and the production modelling variant. Section 3 provides formal results the production variant of the model, and Section 4 for the defence variant of the model. Section 5 looks at an alternative model for the common-enemy hypothesis of by-product mutualism, where players produce a private good instead of a public good. It is shown that heterogeneity does not have any effect on the results here. Section 6 extends the model in the report to multiple players.

**1. List of symbols used**

| $a$ | (degree of) adversity (where in the production model $a=k$, and in the production variant, $a=A$) |
| --- | --- |
| $a_{\min}$ | minimal adversity |
| $a_{\max}$ | maximal adversity |
| $a^{h}$ | adversity such that $\Delta_{1}^{w}\left( a^{h} \right)=\Delta_{0}^{s}(a^{h})$ |
| $A$ | number of attacks in the defence variant |
| $b^{i}\left( x,y \right)$ | benefit of player in role $i=s,w$ when the strong player plays strategy $x$ and the weak player plays strategy $y$ |
| $c$ | cooperation costs |
| $c^{i}$ | cooperation costs of player in role $i=s,w$ |
| $C$ | strategy of cooperating |
| $D$ | strategy of defecting |
| $h$ | (degree of) heterogeneity |
| $k$ | degree of complementarity in the production variant |
| $s$ | indicator for weak player |
| $V$ | value of the collective good when all players cooperate |
| $w$ | indicator for weak player |
| $\Delta_{0}^{i}$ | added benefit of cooperating alone of player in role $i=s,w$ |
| $\Delta_{0}^{i}(.)$ | added benefit of cooperating alone of player in role $i=s,w$ for specific degree of adversity |
| $\Delta_{1}^{i}$ | added benefit of cooperating jointly of player in role $i=s,w$ |
| $\Delta_{1}^{i}(.)$ | added benefit of cooperating jointly of player in role $i=s,w$ for specific degree of adversity |
| $\Delta\left( a^{h} \right)$ | cooperation costs $\Delta\left( a^{h} \right)$ such that ${c=\Delta\left( a^{h} \right)=\Delta}_{1}^{w}\left( a^{h} \right)=\Delta_{0}^{s}(a^{h})$ |

**2. General framework for the results**

Before analysing the separate cases in Sections 3 and 4, we first provide a general framework for describing the results. Specifically, the general version of the results below represents the maximal number of cases that can be obtained for the incidence of the (a)symmetric common-enemy effect and the (a)symmetric deterrence effect (Table 3 in the report), as is the case in Figure 1 in the report (which corresponds to the case in Section 4.2 below); as will be seen in Sections 3 and 4, not all the cases apply to all variants of the model (where all results are stated using the numbering in the general results below, for easy comparison).

Denote by $a_{\min}$ minimal adversity and by $a_{\max}$ maximal adversity; denote by $\Delta_{0}^{s}\left( a_{\min} \right)$, $\Delta_{1}^{w}\left( a_{\min} \right)$, $\Delta_{0}^{s}\left( a_{\max} \right)$ and $\Delta_{1}^{w}\left( a_{\max} \right)$ the values of $\Delta_{0}^{s}$ and $\Delta_{1}^{w}$ corresponding to these extreme degrees of adversity. Then the case in Figure 1(d) (large heterogeneity) of the report is only possible when ${\Delta_{1}^{w}\left( a_{\max} \right)<\Delta}_{0}^{s}\left( a_{\max} \right)$. If instead ${\Delta_{1}^{w}\left( a_{\max} \right)>\Delta}_{0}^{s}\left( a_{\max} \right)$ as in Figures 1(b) and 1(c) of the report (small heterogeneity), then there is a level of adversity $a^{h}$ (with $a_{\min}<a^{h}<a_{\max}$) such that $\Delta_{1}^{w}\left( a^{h} \right)=\Delta_{0}^{s}(a^{h})$, where we denote $\Delta\left( a^{h} \right)=\Delta_{1}^{w}\left( a^{h} \right)=\Delta_{0}^{s}(a^{h})$. We say in these cases that cooperation costs are large when $c>\Delta\left( a^{h} \right)$, and that cooperation costs are small when $c<\Delta\left( a^{h} \right)$. As is clear from Figures 1(b) and 1(c) in the report, we are in the lower range of small heterogeneity when ${\Delta_{1}^{w}\left( a_{max} \right)>\Delta}_{0}^{s}\left( a_{min} \right)$ or $\Delta_{1}^{w}\left( a_{\min} \right)>\Delta_{0}^{s}\left( a_{\max} \right)$, and in the upper range of small heterogeneity when ${\Delta_{1}^{w}\left( a_{max} \right)<\Delta}_{0}^{s}\left( a_{min} \right)$ or $\Delta_{1}^{w}\left( a_{\min} \right)<\Delta_{0}^{s}\left( a_{\max} \right)$. The cases referred to in the general version of the results are those in Table 3 of the report.

**Results (general version):** incidence of (a)symmetric common-enemy and (a)symmetric deterrence effects of higher adversity (Table 3 in the report), as function of cooperation costs $c$ and of heterogeneity $h$:

I. Small heterogeneity (${\Delta_{1}^{w}\left( a_{\max} \right)>\Delta}_{0}^{s}\left( a_{\max} \right)$):

A. Large cooperation costs ($c>\Delta\left( a^{h} \right)$):

(1) Lower range of small heterogeneity (${\Delta_{1}^{w}\left( a_{max} \right)>\Delta}_{0}^{s}\left( a_{min} \right)$):

(i) Upper range of large cooperation costs ($\Delta_{0}^{s}\left( a_{\min} \right)<c<\Delta_{1}^{w}\left( a_{\max} \right)$ ) : symmetric common-enemy effect (Case 1);

(ii) Lower range of large cooperation costs ($\Delta\left( a^{h} \right)<c<\Delta_{0}^{s}\left( a_{\min} \right)$): first asymmetric deterrence, then symmetric common-enemy effect (Case 2).

(2) Upper range of small heterogeneity ($\Delta_{0}^{s}\left( a_{\min} \right)>\Delta_{1}^{w}\left( a_{\max} \right)$:

(i) Upper range of large cooperation costs ($\Delta_{1}^{w}\left( a_{\max} \right)<c<\Delta_{0}^{s}\left( a_{\min} \right)$): asymmetric deterrence effect (Case 5);

(ii) Lower range of large cooperation costs ($\Delta\left( a^{h} \right)<c<\Delta_{1}^{w}\left( a_{\max} \right)$): first asymmetric deterrence, then symmetric common-enemy effect (Case 2).

B. Small cooperation costs ($c<\Delta\left( a^{h} \right)$):

(1) Lower range of small heterogeneity ($\Delta_{1}^{w}\left( a_{\min} \right)>\Delta_{0}^{s}\left( a_{\max} \right)$):

(i) Upper range of small cooperation costs ($\Delta_{1}^{w}\left( a_{\min} \right)<c<\Delta\left( a^{h} \right)$): first asymmetric common-enemy, then symmetric deterrence effect (Case 3);

(ii) Lower range of small cooperation costs ($\Delta_{0}^{s}\left( a_{\max} \right)<c<\Delta_{1}^{w}\left( a_{\min} \right)$): symmetric deterrence effect (Case 4).

(2) Upper range of small heterogeneity ($\Delta_{0}^{s}\left( a_{\max} \right) >\Delta_{1}^{w}\left( a_{\min} \right)$):

(i) Upper range of small cooperation costs ($\Delta_{0}^{s}\left( a_{\max} \right)<c<\Delta\left( a^{h} \right)$): first asymmetric common-enemy, then symmetric deterrence effect (Case 3);

(ii) Lower range of small cooperation costs ($\Delta_{1}^{w}\left( a_{\min} \right)<c<\Delta_{0}^{s}\left( a_{\max} \right)$): asymmetric common-enemy effect (Case 6).

II. Large heterogeneity (${\Delta_{1}^{w}\left( a_{\max} \right)<\Delta}_{0}^{s}\left( a_{\max} \right)$):

A. Large cooperation costs ($\Delta_{0}^{s}\left( a_{\max} \right)<c<\Delta_{0}^{s}\left( a_{\min} \right)$): asymmetric deterrence effect (Case 5);

B. Small cooperation costs ($\Delta_{1}^{w}\left( a_{\min} \right)<c<\Delta_{1}^{w}\left( a_{\max} \right)$): asymmetric common-enemy effect (Case 6).

**4. Production of a public good**

As a benchmark, we first consider the production variant when there is vanishing heterogeneity (De Jaegher and Hoyer, 2016). When two players in a pair cooperate, they produce a public good from which they both obtain benefit $V$ (this means that the public good is non-excludable (Dionisio and Gordo, 2006)), meaning that in Table 1 of the report, $b^{i}\left( C,C \right)=V$ for $i=s,w$; when both players defect, they both obtain benefit $b^{i}\left( D,D \right)=0$ for$i=s,w$. When one player in a pair cooperates and the other player defects, both players obtain benefit $b^{i}\left( C,D \right)=b^{i}\left( D,C \right)=(1- k)V$ for $i=s,w$, where $k$ is the degree of complementarity between the players’ efforts, with $½\leq k\leq1$. It follows that $\Delta_{0}^{s}=\left( 1-k \right)V$ and $\Delta_{1}^{w}=kV$. As an increase in the degree of complementarity decreases the contribution of a first cooperating player compared to a second cooperating player, it makes the impact function relating the value of the public good to the number of cooperating players more convex. A measure treated in the literature that also makes the impact function more convex, is the degree of synergy (Hauert et al., 2006), with the underlying idea that the players’ contributions reinforce each other to a larger extent. Yet, an increase in the degree of synergy shifts the impact function upwards (and therefore cannot be considered as a harsher environment), whereas an increase in the degree of complementarity shifts the impact function downward (and therefore constitutes a harsher environment).

The minimal degree of complementarity we consider is $k=½$, where the players play a linear public goods game ($\Delta_{1}^{w}=\Delta_{0}^{s}=½V$); a first cooperating player contributes value $½V$ to the public good, as does a second cooperating player.^[[1]](#footnote-1)^ When $k=1$, the degree of complementarity is maximal, and the players play a weakest-link public goods game ($\Delta_{1}^{w}\gg\Delta_{0}^{s}$, as $\Delta_{1}^{w}=V$ and $\Delta_{0}^{s}=0$); a first cooperating player contributes nothing to the public good, a second cooperating player, however, contributes the maximal value $V$ to the public good. $k$ thus reflects the extent to which a second cooperating player is pivotal in producing the value of the public good. With the reasoning that each player’s contribution is more pivotal the harsher the environment facing the players, the degree of complementarity $k$ thus serves as a measure for adversity $a$ facing the players, so that $a=k$. It is clear that the degree of complementarity serves as a measure of the harshness of the environment, as it decreases how much value of the public good is produced (where the amount of the public good is not changed when zero or two players cooperate, but is strictly reduced when one player cooperates).

Just as in Figure 1(a) of the report, $\Delta_{0}^{s}$ decreases with adversity, while $\Delta_{1}^{w}$ increases with adversity, with the two added benefits equal for minimal adversity. The symmetric common-enemy effect (switch from the Prisoner’s Dilemma to the Stag Hunt) now applies for large cooperation costs ($c>½V$), and the symmetric deterrence effect (switch from the Harmony Game to the Stag Hunt) applies for small cooperation costs ($c<½V$). We now consecutively check to what extent these results are maintained in versions of the production variant of the model where players are heterogeneous in their capability to contribute to the public good, in the benefits they obtain from the public good, and in their cooperation costs.

*4.1. Heterogeneity in capability to contribute to the public good*

We assume here that all players obtain the same benefit from the public good ($b^{i}\left( x,y \right)=b^{j}\left( x,y \right)$ for $i\neq j$, $x,y=C,D$), but have a different capability of contributing to the public good. It is the case that $b^{s}\left( C,C \right)=b^{w}\left( C,C \right)=V$ and $b^{s}\left( D,D \right)=b^{w}\left( D,D \right)=0$, but $b^{i}\left( C,D \right)>b^{i}\left( D,C \right)$ for $i=s,w$. When the strong player cooperates and the weak player defects, $b^{s}\left( C,D \right)=b^{w}\left( C,D \right)=2\left( 1-k \right)hV$. When the weak player cooperates and the strong player defects,$b^{s}\left( D,C \right)=b^{w}\left( D,C \right)=2\left( 1-k \right)(1-h)V$. It continues to be the case that $k$ reflects the degree of complementarity, with $½\leq k\leq1$. The parameter $h$, with $½\leq h\leq1$, now reflects the extent to which the players have heterogeneous capabilities of contributing to the public good, where the larger $h$, the more the strong player contributes compared to the weak player; when $h=½$, we have vanishing heterogeneity as treated above as a benchmark, when $h=1$, heterogeneity is maximal and when the weak player cooperates alone, this does not contribute anything to the public good. It is easy to check that $\Delta_{0}^{s}$ and $\Delta_{1}^{w}$ equal:

$\Delta_{0}^{s}=2\left( 1-k \right)hV$ (S1)

$\Delta_{1}^{w}=\left[ 1-2\left( 1-k \right)h \right]V$ (S2)

Following the general version of the results in Section 2 above, the critical added benefits determining the incidence of the different cases are (with $a=k)$:

$\Delta_{0}^{s}\left( k_{\min} \right)=hV$ (S3)

$\Delta_{1}^{w}\left( k_{\min} \right)=(1-h)V$ (S4)

$\Delta_{0}^{s}\left( k_{\max} \right)=0$ (S5)

$\Delta_{1}^{w}\left( k_{\max} \right)=V$ (S6)

$\Delta\left( k^{h} \right)=½V$, with $k^{h}=(4h-1)/(4h)$ (S7)

Given that the cases in the general version of the results in Section 2 are determined by the relation of the critical added benefits (S3)-(S7) to each other and to the cooperation costs, Figure S1 represents these critical added benefits as a function of $h$. Clearly, for $h=½$, $\Delta_{0}^{s}\left( k_{\min} \right)=\Delta\left( k^{h} \right)=\Delta_{1}^{w}\left( k_{\min} \right)=½V$, $\Delta_{1}^{w}\left( k_{\max} \right)=V$, and $\Delta_{0}^{s}\left( k_{\max} \right)=0$; for $h=1$, $\Delta_{0}^{s}\left( k_{\min} \right)=\Delta_{1}^{w}\left( k_{\max} \right)=V$, and $\Delta_{1}^{w}\left( k_{\min} \right)=\Delta_{0}^{s}\left( k_{\max} \right)=0$. Also, $\Delta_{0}^{s}\left( k_{\max} \right)$, $\Delta_{1}^{w}\left( k_{\max} \right)$ and $\Delta\left( k^{h} \right)$ are flat as a function of $h$, and $\Delta_{0}^{s}\left( k_{\min} \right)$ is increasing in $h$, while $\Delta_{1}^{w}\left( k_{\min} \right)$ is decreasing in $h$.


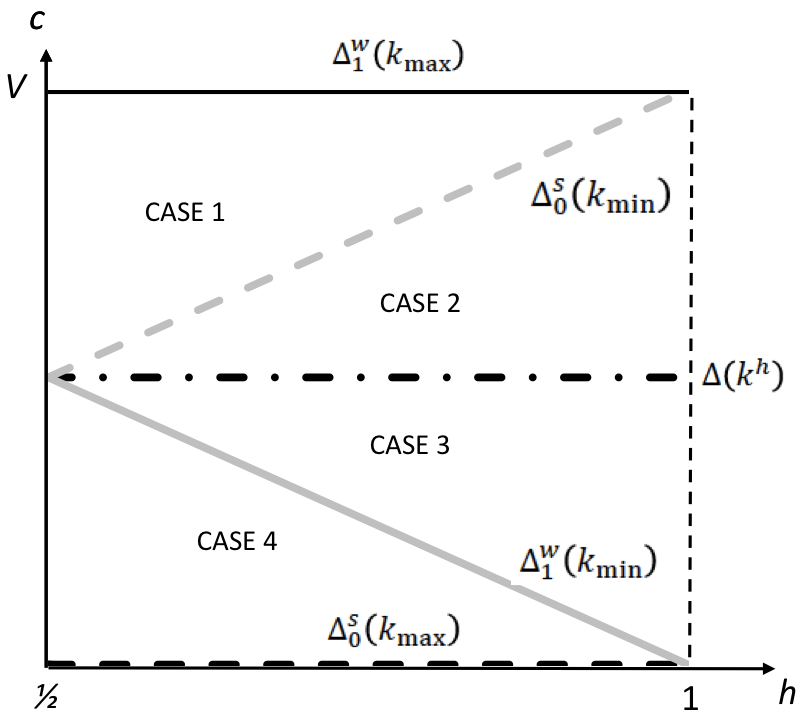


**Fig. S1. Production variant with heterogeneous capability to contribute to the public good**, where adversity is measured by the degree of complementarity ($a=k$). Critical added benefits as a function of heterogeneity $h$. As a function of the relation of cooperation costs to these critical added benefits, the different cases for the effect of higher adversity on the probability of cooperation (Table 3 of the report) are indicated: Case 1: symmetric common-enemy effect; Case 2: first asymmetric deterrence effect, then symmetric common-enemy effect; Case 3: first asymmetric common-enemy effect, then symmetric deterrence effect; Case 4: symmetric deterrence effect.

We can now in Figure S1 indicate for each combination of $c$ and $h$ which case in Table 3 of the report applies. As it is always the case that ${\Delta_{1}^{w}\left( k_{\max} \right)>\Delta}_{0}^{s}\left( k_{\max} \right)$ (small heterogeneity), and that $\Delta_{0}^{s}\left( k_{\min} \right)<\Delta_{1}^{w}\left( k_{\max} \right)$ and $\Delta_{1}^{w}\left( k_{\min} \right)>\Delta_{0}^{s}\left( k_{\max} \right)$ when $h<1$ (lower range of small heterogeneity), the cases of large heterogeneity, and of the upper range of small heterogeneity in the general version of the results in Section 2 are irrelevant for this modelling variant. This shows that Cases 5 and 6 of Table 3 in the report never occur in this variant, so that only Figure 1(b) of the report is relevant. The reason for this is that large complementarity undoes the effects of heterogeneity: when each player’s cooperative effort is critical for producing the public good (maximal complementarity), the fact that they contribute heterogeneously to the public good loses its significance. In other words, as long as complementarity is sufficiently large, heterogeneity will be weak relatively speaking. For this reason, for sufficiently large complementarity, it is always the case that the symmetric effects of higher adversity in Table 3 of the report apply. These results are summarised in the following adjusted version of the general results in Section 2.

**Results (version for the production variant with** **heterogeneous capacity to contribute to the public good):** incidence of cases in Table 3 of the report for the effect of higher adversity, as function of cooperation costs $c$ and of heterogeneity $h$:

I. $h<1$:

A. $½V<c<V$: (large $c$):

(1) (i) $hV<c<V$ (upper range of large $c$): Case 1;

(ii) $½V<c<hV$ (lower range of large $c$): Case 2.

B. $c<½V$ (small $c$):

(1) (i) $\left( 1-h \right)V<c<½V$ (upper range of small $c$): Case 3;

(ii) $0<c<\left( 1-h \right)V$ (lower range of small $c$): Case 4.

As can be seen from Figure S1, Cases 2 and 3 of Table 3 in the report apply for a larger range of cooperation costs the larger heterogeneity, where for heterogeneity approaching its maximal level, Cases 1 and 4 vanish. Moreover, within Case 2 and Case 3, as $k^{h}=(4h-1)/(4h)$ increases in $h$, the asymmetric effects from Table 3 of the report apply for a wider range of degrees of complementarity as $h$ increases, where as $h$ approaches ½, they never apply, and as $h$ approaches 1, they apply whenever $k<¾$, and thus for exactly half the range of degrees of complementarity.

*4.2 Heterogeneity in value obtained from the public good*

We now assume that players contribute the same value to the public good, meaning that $b^{i}\left( C,D \right)=b^{i}\left( D,C \right)$ for $i=s,w$ (for the value obtained from the public good, what matters is how many players cooperate, where it does not matter whether the cooperating player is the weak or the strong player). Yet, we assume that players obtain different values from the public good, i.e. $b^{s}\left( x,y \right)>b^{w}\left( x,y \right)$ for $x,y=C,D$. To keep this case comparable with the benchmark case with vanishing heterogeneity, we assume that the total value produced of the public good is $2V$ when both players cooperate, $2(1-k)V$ if one player cooperates, and 0 when no player cooperates. Of these values, the strong player obtains a share $h$, and the weak player obtains a share $(1-h)$, with $h\geq½$. In this way, for $h=½$ (each obtains half of the produced value of the public good), we obtain the benchmark case with vanishing heterogeneity. We therefore have $b^{s}\left( C,C \right)=h2V$, $b^{s}\left( C,D \right)=b^{s}\left( D,C \right)=h2\left( 1-k \right)V$, $b^{s}\left( D,D \right)=0$; also, $b^{w}\left( C,C \right)=(1-h)2V$, $b^{w}\left( C,D \right)=b^{w}\left( D,C \right)=(1-h)2\left( 1-k \right)V$, $b^{w}\left( D,D \right)=0$. It is the case that:

$\Delta_{0}^{s}=h2\left( 1-k \right)V$ (S8)

$\Delta_{1}^{w}=\left( 1-h \right)2kV$ (S9)

Following again the general version of the results in Section 2, the critical added benefits determining the incidence of the different cases in Table 3 of the report are (with $a=k$):

$\Delta_{0}^{s}\left( k_{\min} \right)=hV$ (S10)

$\Delta_{1}^{w}\left( k_{\min} \right)=(1-h)V$ (S11)

$\Delta_{0}^{s}\left( k_{\max} \right)=0$ (S12)

$\Delta_{1}^{w}\left( k_{\max} \right)=2(1-h)V$ (S13)

$\Delta\left( k^{h} \right)=2h\left( 1-h \right)V$, with $k^{h}=h$ (S14)

We note now that for $h=½$, it is the case that $\Delta_{0}^{s}\left( k_{\min} \right)=\Delta\left( k^{h} \right)=\Delta_{1}^{w}\left( k_{\min} \right)=½V$, that $\Delta_{0}^{s}\left( k_{\max} \right)=0$, and that $\Delta_{1}^{w}\left( k_{\max} \right)=V$. For $h=1$, it is the case that $\Delta_{1}^{w}\left( k_{\min} \right)=\Delta\left( k^{h} \right)=\Delta_{1}^{w}\left( k_{\max} \right)=0$, that $\Delta_{0}^{s}\left( k_{\max} \right)=0$, and that $\Delta_{0}^{s}\left( k_{\min} \right)=V$. Clearly, in Figure S2, for $h<1$, $\Delta_{1}^{w}\left( k_{\max} \right)$ lies everywhere above $\Delta_{1}^{w}\left( k_{\min} \right)$. Also, in Figure S2, $\Delta\left( k^{h} \right)$ is a concave decreasing function that lies between $\Delta_{1}^{w}\left( k_{\min} \right)$ and $\Delta_{1}^{W}\left( k_{\max} \right)$. Furthermore, it is the case that $\Delta_{0}^{s}\left( k_{\min} \right)⋛\Delta_{1}^{w}\left( k_{\max} \right)$ when $h⋛⅔$. Also, it is the case that$\Delta_{1}^{w}\left( k_{\min} \right)>\Delta_{0}^{s}\left( k_{\max} \right)$ when $h<1$. Finally, for $h<1$, it is always the case that ${\Delta_{1}^{w}\left( k_{\max} \right)>\Delta}_{0}^{s}\left( k_{\max} \right)$.

These conclusions allow us to represent in Figure S2 what cases from Table 3 in the report apply for different levels of $c$ and $h$. Using the general version of the results in Section 2, as it is always the case that ${\Delta_{1}^{w}\left( k_{\max} \right)>\Delta}_{0}^{s}\left( k_{\max} \right)$ (small heterogeneity), it follows that the case of large heterogeneity, where only Case 5 and Case 6 (Table 3 in the report) are relevant, never occurs for this variant of the model (meaning that the scenario in Figure 1(d) in the report is never relevant). The cost level $\Delta\left( k^{h} \right)$ that delineates the cases of small and large cooperation costs, namely $2h\left( 1-h \right)V$, depends on the level of $h$, in such a way that the case of small cooperation costs applies for a smaller and smaller range of costs as heterogeneity is increased. Finally, for large cooperation costs ($c>2h\left( 1-h \right)V$), for $h>⅔$, we switch to the upper range of small heterogeneity, where the relevant cases from Table 3 in the report are Case 5 and Case 6 (meaning that for large cooperation costs, both Figures 1(b) and 1(c) in the report are relevant). For small cooperation costs, ($c<2h\left( 1-h \right)V$), what is referred to in the general version of the results in Section 2 as the lower range of small heterogeneity, is the only relevant case of heterogeneity, so that the only two relevant cases from Table 3 in the report, whatever the level of heterogeneity, are Cases 3 and 4 (meaning that for small cooperation costs, only Figure 1(b) in the report is relevant). Summarising:

**Results (version for the production variant with** **heterogeneous values obtained from the public good):** incidence of cases in Table 3 of the report for the effect of higher adversity, as function of cooperation costs $c$ and of heterogeneity $h$:

I. $h<1$:

A.$c>2\left( 1-h \right)hV$ (large $c$):

(1) $h<⅔$ (lower range of small $h$):

(i) $2\left( 1-h \right)V<c<hV$ (upper range of large $c$): Case 1;

(ii) $2\left( 1-h \right)hV<c<2\left( 1-h \right)V$ (lower range of large $c$): Case 2.

(2) $⅔<h<1$ (upper range small of $h$):

(i) $hV<c<2\left( 1-h \right)V$ (upper range of large $c$): Case 5;

(ii) $2\left( 1-h \right)hV<c<hV$ (lower range of large $c$): Case 2.

B. $c<2\left( 1-h \right)hV$ (small $c$):

(1) (i) $\left( 1-h \right)V<c<2\left( 1-h \right)hV$ (upper range of small $c$): Case 3;

(ii) $0<c<\left( 1-h \right)V$ (lower range of small $c$): Case 6.

The reason for the asymmetry in the results is that in terms of Figure 1(b) in the report, because the weak player gets less and the strong player more, as a function of adversity, $\Delta_{1}^{w}$ is rather flat, and $\Delta_{0}^{s}$ is rather steep. For this reason, as increased heterogeneity shifts these curves, the relation between $\Delta_{1}^{w}\left( k_{\min} \right)$ and $\Delta_{0}^{s}\left( k_{\max} \right)$ does not change, and for this reason Case 6 in Table 3 of the report is never obtained. Intuitively, for large complementarity, the impact of heterogeneity disappears for $\Delta_{0}^{s}$ (if a first player contributes vanishingly little, it does not matter which share of the public good he obtains; $\Delta_{0}^{s}=0$ for $k=1$, whatever $h$), but does not disappear for $\Delta_{1}^{w}$ (as the full value of the public good is always obtained if both players cooperate, it does matter what share of the public good one obtains; $\Delta_{1}^{w}<V$ for $k=1$ as soon as $h>½$); this explains why for small cooperation costs as complementarity is increased, eventually one obtains a symmetric effect, while for large cooperation costs this is not the case.


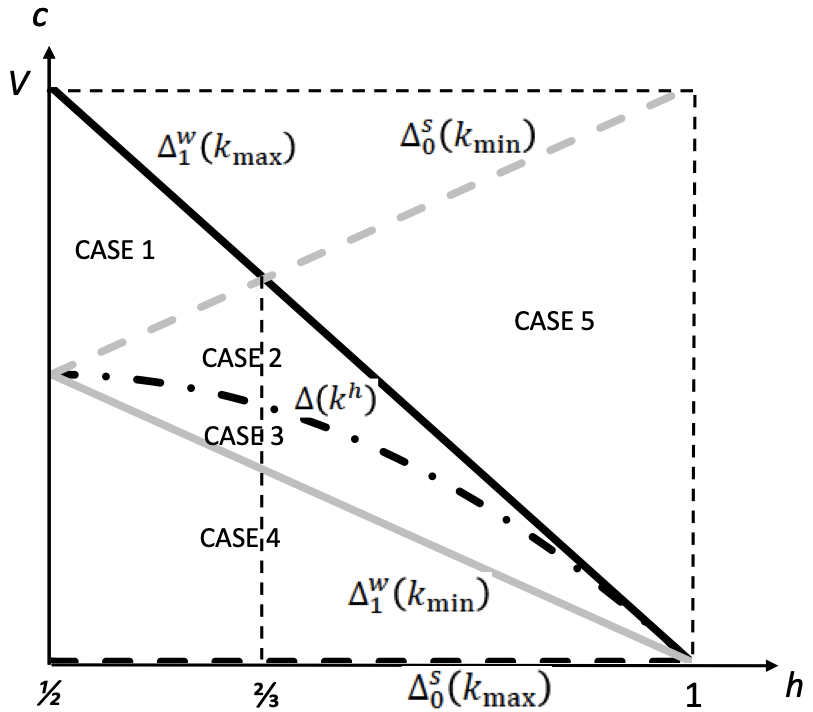


**Fig. S2. Production variant with heterogeneous values obtained from the public good**, where adversity is measured by the degree of complementarity ($a=k$). Critical added benefits as a function of heterogeneity $h$. In line with Result 2 in the general version of the Results in Section 2, the different cases for the effect of higher adversity (see Table 3 in the report) are indicated. Case 1: symmetric common-enemy effect; Case 2: first asymmetric deterrence effect, then symmetric common-enemy effect; Case 3: first asymmetric common-enemy effect, then symmetric deterrence effect; Case 4: symmetric deterrence effect; Case 5: asymmetric deterrence effect.

We further explain why, as illustrated in Figure S2, Case 5 of Table 3 in the report becomes predominant as heterogeneity is increased. If heterogeneity is increased, this means moving closer to a situation where, even if cooperation costs are small, only the strong player can benefit from cooperating (as he obtains most of the value of the public good), leading to a Free-Rider game as long as the degree of complementarity is not too large. If one now additionally increases the degree of complementarity, one moves to a situation where a single cooperating player means a low value of the public good; as heterogeneity here means obtaining different shares of the public good, large complementarity does not undo the effect of heterogeneity, and as complementarity is increased the game becomes a Prisoner’s Dilemma.

We end the analysis of this variant by looking at how, within Cases 2 and 3 of Table 3 in the report, the relevance of the asymmetric effects is affected by the level of heterogeneity. As $k^{h}=h$ (see (S14)), the asymmetric part of the effects in Table 3 of the report applis for a wider range of small degrees of complementarity as $h$ increases, where as $h$ approaches ½, they never apply, and as $h$ approaches 1, they apply for all degrees of complementarity.

*4.3 Heterogeneity in cooperation costs*

We now consider a version of the production variant of the model where benefits are homogeneous (meaning that $b^{i}\left( x,y \right)=b^{j}\left( x,y \right)$, and $b^{i}\left( x,y \right)=b^{i}\left( y,x \right)$ for $x,y=C,D$), but where a strong player incurs cooperation costs $c^{s}=2(1-h)c$, whereas a weak player incurs cooperation costs $c^{w}=2hc$, with $½ \leq h\leq1$, and where average cooperation costs within a heterogeneous pair equal $½\left( 2hc \right)+½\left( 2\left( 1-h \right)c \right)=c$. As only the relation between added benefits and cooperation costs matters for the results, we consider the players as having the same average cooperation costs $c$, but having different added benefits, where these added benefits are adjusted to take into account the heterogeneity in costs. Contrary to what is the case with the other forms of heterogeneity, this representation leads to added benefits that are non-linear in the degree of heterogeneity.

$\Delta_{0}^{s}=\left( 1-k \right)V/[2(1-h)]$ (S15)

$\Delta_{1}^{w}=kV/(2h)$ (S16)

Following the general version of the Results in Section 2, the critical added benefits equal (with $a=k)$:

$\Delta_{0}^{s}\left( k_{\min} \right)=V/[4\left( 1-h \right)]$ (S17)

$\Delta_{1}^{w}\left( k_{\min} \right)=V/(4h)$ (S18)

$\Delta_{0}^{s}\left( k_{\max} \right)=0$ (S19)

$\Delta_{1}^{w}\left( k_{\max} \right)=V/(2h)$ (S20)

$\Delta\left( k^{h} \right)=½V$, with $k^{h}=h$ (S21)

These critical added benefits are depicted in Figure S3 as a function of $h$. For $h=½$, it is the case that $\Delta_{0}^{s}\left( k_{\min} \right)=\Delta\left( k^{h} \right)=\Delta_{1}^{w}\left( k_{\min} \right)=½V$, that $\Delta_{0}^{s}\left( k_{\max} \right)=0$, and that $\Delta_{1}^{w}\left( k_{\max} \right)=V$. For $h=1$, it is the case that $\Delta_{1}^{w}\left( k_{\min} \right)=¼V$, and that $\Delta_{1}^{w}\left( k_{\max} \right)=\Delta\left( k^{h} \right)=½V$. Also, $\Delta_{0}^{s}\left( k_{\min} \right)=V$ when $h=¾$; $\Delta_{0}^{S}\left( k_{\min} \right)$ itself is an increasing convex function of $h$. Furthermore, $\Delta_{1}^{w}\left( k_{\max} \right)$ lies everywhere above $\Delta_{1}^{w}\left( k_{\min} \right)$, where both are convex decreasing functions of $h$. Also, for $½<h<1$, $\Delta\left( k^{h} \right)$ lies between $\Delta_{1}^{w}\left( k_{\min} \right)$ and $\Delta_{1}^{w}\left( k_{\max} \right)$, and also lies between $\Delta_{1}^{w}\left( k_{\min} \right)$ and $\Delta_{0}^{s}\left( k_{\min} \right)$. Furthermore, it is the case that $\Delta_{0}^{s}\left( k_{\min} \right)⋛\Delta_{1}^{w}\left( k_{\max} \right)$ when $h⋛2/3$, where $\Delta_{0}^{s}\left( k_{\min} \right)=\Delta_{1}^{w}\left( k_{\max} \right)=¾V$ when $h=2/3$. Finally, it is always the case that$\Delta_{1}^{w}\left( k_{\min} \right)>\Delta_{0}^{s}\left( k_{\max} \right)$.


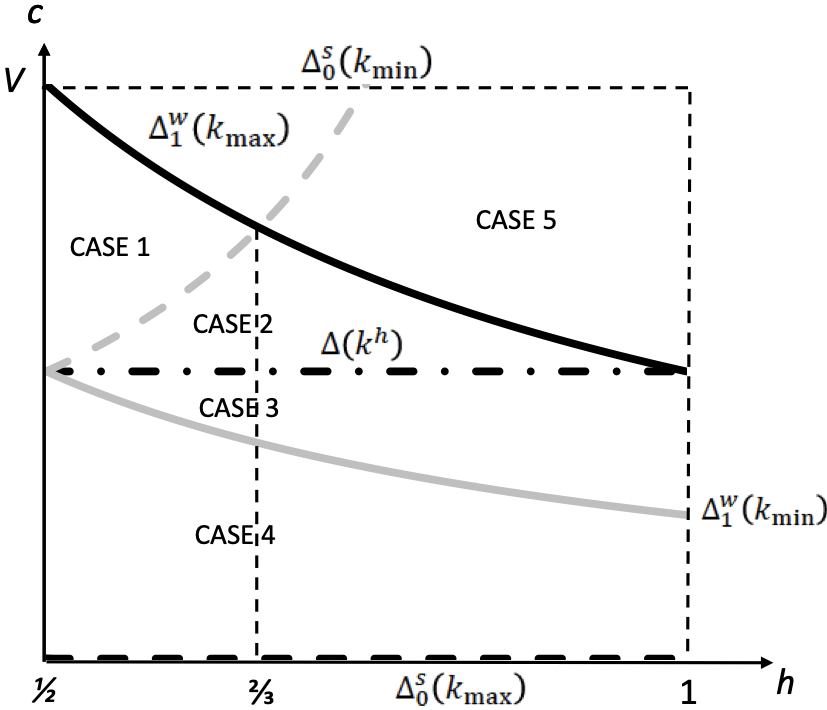


**Fig. S3.** Production variant with heterogeneous cooperation costs, where adversity is measured by the degree of complementarity ($a=k$). Critical added benefits (as a function of heterogeneity $h$. In line with the general version of the Results in Section 2, the different cases for the effect of higher adversity (see Table 3 in the report) are indicated. Case 1: symmetric common-enemy effect; Case 2: first asymmetric deterrence effect, then symmetric common-enemy effect; Case 3: first asymmetric common-enemy effect, then symmetric deterrence effect; Case 4: symmetric deterrence effect; Case 5: asymmetric deterrence effect.

As it is always the case that ${\Delta_{1}^{w}\left( k_{\max} \right)>\Delta}_{0}^{s}\left( k_{\max} \right)$, the case of large heterogeneity, where only Case 5 and Case 6 in Table 3 of the report are relevant, never occurs for the production variant with heterogeneous costs (meaning that scenarios such as in Figure 1(d) in the report are never relevant). Independently of the degree of heterogeneity, cooperation costs are large when $c>½V$ and small when $c<½V$. For large cooperation costs, given that $\Delta_{0}^{s}\left( k_{\min} \right)⋛\Delta_{1}^{w}\left( k_{\max} \right)$ when $h⋛⅔$, Cases 1 and 2 of Table 3 in the report are relevant when $h<⅔$ (lower range of small heterogeneity), and Cases 2 and 5 when $h>⅔$ (upper range of small heterogeneity). For small cooperation costs, given that $\Delta_{1}^{w}\left( k_{\min} \right)>\Delta_{0}^{s}\left( k_{\max} \right)$, only the case indicated as the lower range of small heterogeneity in the general version of the results in Section 2 is relevant, and the relevant cases are Case 3 and Case 4. These conclusions are summarised in the adjusted version of the general version of the results in Section 2 for this case:

**Results (version for the production variant with heterogeneous costs):** incidence of cases in Table 3 of the report for the effect of higher adversity, as function of cooperation costs $c$ and of heterogeneity $h$:

I. $h<1$:

A. $c>½V$ (large $c$):

(1) $h<2/3$ (lower range of small $h$):

(i) $V/[4\left( 1-h \right)]<c<V/(2h)$ (upper range of large $c$): Case 1;

(ii) $½V<c<V/[4\left( 1-h \right)]$ (lower range of large $c$): Case 2.

(2) $⅔<h<1$ (upper range of large $h$):

(i) $V/(2h)<c<V/[4\left( 1-h \right)]$ (upper range of large $c$): Case 2*;

(ii) $½V<c<V/(2h)$ (lower range of large $c$): Case 2.

B. $c<½V$ (small $c$):

(1) (i) $V/(4h)<c<V/2$ (upper range of small $c$): Case 3;

(ii) $c<V/(4h)$ (lower range of small $c$): Case 4.

The results are similar to those with heterogeneous values obtained from the public good (Section 3.2), which can be understood by the fact that a model where players put different values on the public good, should lead to similar results as a model where players have different costs of contributing to the public good; indeed, this is witnessed by the fact that having heterogeneous cooperation costs can be represented by having heterogeneous adjusted benefits. Yet, a difference with the results for heterogeneous values obtained from the public good is that Case 5 of Table 3 in the report does not become predominant as complementarity is increased. The reason for this is that, with maximal heterogeneity, where the weak player bears the entire average cooperation costs, the weak player continues to benefit from the public good.

**4. Defence of a public good**

We next treat a variant of the model where players jointly defend an existing public good, and consider the case with vanishing heterogeneity (De Jaegher and Hoyer (2016)) as a benchmark. This variant represents in a stylised way a situation of cycloalexy, or circular defence (Jolivet at al., 1990). Each player in a group of $n$ players is positioned on one of the $n$ sides of a common territory. The players face a number of random attacks $A$, where $A$ is now our measure of adversity, so that in terms of the framework in the report, $a=A$ (where $A_{\min}=1$, and $A_{\max}=+\infty$). Random attacks, when expressed as a statistical process, take the form of sampling with replacement of the $n$ sides of the common territory. With $n=2$, it follows that with probability, $1/2^{A}$, only one specific player’s side is attacked (or in short: only one player is attacked), and with probability $[1-2/2^{A}]$, both attackers’ sides are attacked at least once (or in short: both players are attacked).

Cooperating ($C$) now means doing effort to defend one’s side (which comes again at cooperation costs $c$), defecting ($D$) means not doing effort. When a player’s side is not attacked, then the value of the common territory is not affected by whether or not this player defends. When a player’s side is attacked one or more times and the player defends, the attacks on this player do not affect the value of the common territory, so that a defending player is able to fight off any number of attacks directed at his side. The value of the common territory is only reduced when at least one player is attacked who does not defend. In this sense, a player who does not defend (i.e. defects), but is never attacked, can be seen as still contributing to the defence of the common territory, and we make a distinction between whether a player takes defensive effort or not, and whether a player contributes or does not contribute to the defence of the common territory. If both players contribute to the defence of the common territory (either because they both take defensive efforts, or because only one player defends but only he is attacked), they both obtain benefit $V$ from the common territory. If neither player contributes (which occurs only if neither player takes defensive efforts, and they are both attacked), both players obtain benefit 0 from the common territory. If one player contributes and the other does not (where the latter player does not take defensive effort and is attacked, and where the former player either is not attacked, or is attacked but took defensive effort), both players obtain benefit $(1- k)V$ from the common territory, where $k$ is the degree of complementarity between the players’ contributions, which is now considered as given. In the extreme case where $k=1$, the entire common territory is lost as soon as one non-defending player is attacked at least once; for smaller values of $k$, some value of the common territory is maintained in these circumstances.

Specifically in terms of Table 1 in the report, if both players cooperate, their payoffs do not depend on whether or not they are attacked, and they both obtain benefit $V$ from the common territory, so that $b^{i}\left( C,C \right)=V$ for $i=s,w$. If one player cooperates and the other defects, with probability $1/2^{A}$ only the cooperating player is attacked and both players again obtain benefit $V$ from the common territory; with the complementary probability, the defecting player is attacked at least once, and both players obtain benefit $(1- k)V$ from the common territory; it follows that in Table 1 of the report, $b^{i}\left( C,D \right)=b^{i}\left( D,C \right)=\frac{1}{2^{A}}V+\left[ 1-\frac{1}{2^{A}} \right]\left( 1- k \right)V$ for $i=s,w$. If both players defect, then with probability $2/2^{A}$, only a single player is attacked, meaning that both players obtain benefit $(1- k)V$ from the common territory; with the complementary probability they are both attacked, and they obtain a zero benefit from the common territory; it follows that $b^{i}\left( D,D \right)=\frac{2}{2^{A}}V(1-k)$ for$i=s,w$. The added benefits can now be calculated as:

$\Delta_{0}^{s}=\frac{1}{2^{A}}(2k-1)V+\left[ 1-\frac{1}{2^{A}} \right]\left( 1- k \right)V$ (S22)

$\Delta_{1}^{w}=\left[ 1-\frac{1}{2^{A}} \right]kV$ (S23)

It is easily checked that $\Delta_{0}^{s}=\Delta_{1}^{w}=½kV$ for $A=1$, and that $\Delta_{1}^{w}$ increases in $A$, whereas $\Delta_{0}^{s}$ decreases in $A$ as long as $k>⅔$. Thus, assuming $k>⅔$, for large cooperation costs $c>½kV$ we have a symmetric common-enemy effect of an increase in the number of random attacks, and for small cooperation costs $c<½kV$ we have a symmetric deterrence effect of such an increase. Intuitively, an increase in the number of random attacks in the defence variant, has a similar effect to an increase in complementarity in the production variant. Such a comparison makes sense, as players in the defence variant may be seen as producing collective defence, where a larger number of attacks makes each player’s effort more critical. As $A$ approaches infinity, $\Delta_{0}^{s}$ approaches $(1-k)V$ and $\Delta_{1}^{w}$ approaches $kV$, meaning that for large $A$, we obtain a case equivalent to the production variant with degree of complementarity $k$. Thus, increases in the number of attacks are equivalent to an increase in complementarity in the production variant, only if $k$ is sufficiently large (the fact that it does not suffice for this equivalence that $k>½$, has to do with the fact that in the defence variant, $b^{i}\left( D,D \right)$ is not typically equal to zero).

We now again check to what extent these results are maintained in versions of the defence variant where players have either heterogeneous capabilities to contribute to collective defence, obtain heterogeneous shares of the common territory defended, or have heterogeneous costs.

*4.1 Heterogeneity in capability to contribute to the public good*

In the same manner as for the variant with production of a public good, we first assume that all players have a different capability of contributing to the public good. Just as in the case, with vanishing heterogeneity we have $b^{s}\left( C,C \right)=b^{w}\left( C,C \right)=V$ and $b^{s}\left( D,D \right)=b^{w}\left( D,D \right)$ $=\frac{1}{2^{A}}2\left( 1-k \right)hV+\frac{1}{2^{A}}2\left( 1-k \right)(1-h)V$ $=\frac{2}{2^{A}}\left( 1-k \right)V$. When the strong player cooperates and the weak player defects, $b^{s}\left( C,D \right)=b^{w}\left( C,D \right)=\frac{1}{2^{A}}V+\left[ 1-\frac{1}{2^{A}} \right]2\left( 1- k \right)hV$. When the weak player cooperates and the strong player defects,$b^{s}\left( D,C \right)=b^{w}\left( D,C \right)=\frac{1}{2^{A}}V+\left[ 1-\frac{1}{2^{A}} \right]2\left( 1- k \right)(1-h)V$. One can now check that $\Delta_{0}^{s}$ and $\Delta_{1}^{w}$ equal:

$\Delta_{0}^{s}=\frac{1}{2^{A}}[1-2(1-k)]V+\left[ 1-\frac{1}{2^{A}} \right]2\left( 1- k \right)hV$ (S24)

$\Delta_{1}^{w}=\left[ 1-\frac{1}{2^{A}} \right][1-2\left( 1-k \right)h]V$ (S25)

For ease of exposition, we consider $A$ as a real number, rather than an integer. It is the case that $\frac{{\partial\Delta}_{0}^{s}}{\partial A}<0$ as long as $k>(1+2h)/[2(1+h)]$. For $h$ approaching 1, this condition becomes $k>¾$. The general version of the results in Section 2 applies for all possible levels of heterogeneity, as long as $k>¾$ (sufficiently large complementarity). Following this framework, the critical added benefits determining the incidence of the different cases (with $a=A$), which are represented in Figure S4, are:

$\Delta_{0}^{s}\left( A_{\min} \right)=½[1-2\left( 1-k \right)(1-h)]V$ (S26)

$\Delta_{1}^{w}\left( A_{\min} \right)=½[1-2\left( 1-k \right)h]V$ (S27)

$\Delta_{0}^{s}\left( A_{\max} \right)=2\left( 1- k \right)hV$ (S28)

$\Delta_{1}^{w}\left( A_{\max} \right)=[1-2\left( 1-k \right)h]V$ (S29)

$\Delta\left( A^{h} \right)=\frac{2k-1}{2[k-2\left( 1-k \right)h]}[1-2\left( 1-k \right)h]V$, with $A^{h}=\ln\left\{ \frac{2\left[ k-2\left( 1-k \right)h \right]}{1-4\left( 1-k \right)h} \right\}/[\ln(2)]$

(S30)

Note that for $k=1$, all effects of heterogeneity are undone, with $\Delta\left( A^{h} \right)=½V$, $A^{h}=1$, $\Delta_{0}^{s}\left( A_{\min} \right)=\Delta_{1}^{w}\left( A_{\min} \right)=½V$, $\Delta_{0}^{s}\left( A_{\max} \right)=0$, $\Delta_{1}^{w}\left( A_{\max} \right)=V$. The cases of interest are thus ones where $¾<k<1$. For $h=½$, $\Delta_{0}^{s}\left( A_{\min} \right)=\Delta\left( A^{h} \right)=\Delta_{1}^{w}\left( A_{\min} \right)=½kV$, $\Delta_{1}^{w}\left( A_{\max} \right)=kV$, and $\Delta_{0}^{s}\left( A_{\max} \right)=\left( 1-k \right)V$, where $\Delta_{0}^{s}\left( A_{\max} \right)<\Delta_{1}^{w}\left( A_{\min} \right)=\Delta\left( A^{h} \right)=\Delta_{0}^{s}\left( A_{\min} \right)<\Delta_{1}^{w}\left( A_{\max} \right)$. For $h=1$,$\Delta_{0}^{s}\left( A_{\min} \right)=½V$, $\Delta_{1}^{w}\left( A_{\min} \right)=½(2k-1)V$, $\Delta_{0}^{s}\left( A_{\max} \right)=2\left( 1- k \right)V$, $\Delta_{1}^{w}\left( A_{\max} \right)=(2k-1)V$, $\Delta\left( A^{h} \right)=\frac{{(2k-1)}^{2}}{2[3k-2]}V$. It can be checked that for $h=1$ is the case that $\Delta_{1}^{w}\left( A_{\min} \right)<\Delta_{0}^{s}\left( A_{\max} \right)<\Delta\left( A^{h} \right)<\Delta_{0}^{s}\left( A_{\min} \right)<\Delta_{1}^{w}\left( A_{\max} \right)$ when $¾<k<⅚$, and $\Delta_{0}^{s}\left( A_{\max} \right)<\Delta_{1}^{w}\left( A_{\min} \right)<\Delta\left( A^{h} \right)<\Delta_{0}^{s}\left( A_{\min} \right)<\Delta_{1}^{w}\left( A_{\max} \right)$ when $⅚<k<1$. Figure S4 represents an example where $k>⅚$.

For $h>½$, it is the case that $\Delta_{1}^{w}\left( A_{\min} \right)<\Delta_{0}^{s}\left( A_{\min} \right)$, with $\Delta_{0}^{s}\left( A_{\min} \right)$ increasing in $h$, and $\Delta_{1}^{w}\left( A_{\min} \right)$ decreasing in $h$. As $\Delta\left( A^{h} \right)$ is concave and increasing in $h$, and as for $h=1$ it is the case that $\Delta\left( A^{h} \right)<\Delta_{0}^{s}\left( A_{\min} \right)$, it follows that for $h>½$,$\Delta\left( A^{h} \right)$ lies between $\Delta_{1}^{w}\left( A_{\min} \right)$ and $\Delta_{0}^{s}\left( A_{\min} \right)$. Also, for any $h$ it is the case that $\Delta_{0}^{s}\left( A_{\max} \right)<\Delta_{0}^{s}\left( A_{\min} \right)$, and $\Delta_{0}^{s}\left( A_{\max} \right)$ is an increasing function of $h$. Given that $\Delta_{0}^{s}\left( A_{\max} \right)<\Delta\left( A^{h} \right)=\Delta_{0}^{s}\left( A_{\min} \right)$ for $h=½$, that $\Delta_{0}^{s}\left( A_{\max} \right)<\Delta\left( A^{h} \right)<\Delta_{0}^{s}\left( A_{\min} \right)$ for $h=1$, and that $\Delta\left( A^{h} \right)$ is concave and increasing, it follows that for $h>½$,$\Delta\left( A^{h} \right)$ lies between $\Delta_{0}^{s}\left( A_{\max} \right)$ and $\Delta_{0}^{s}\left( A_{\min} \right)$. As $\Delta_{0}^{s}\left( A_{\min} \right)<\Delta_{1}^{w}\left( A_{\max} \right)$ both for $h=½$ and for $h=1$, and as $\Delta_{1}^{w}\left( A_{\max} \right)$ is monotonically decreasing in $h$, it follows that $\Delta_{0}^{s}\left( A_{\min} \right)<\Delta_{1}^{w}\left( A_{\max} \right)$ for all $h$. Finally, for $⅚<k<1$, it is the case for all $h$ that $\Delta_{0}^{s}\left( A_{\max} \right)<\Delta_{1}^{w}\left( A_{\min} \right)$; for $¾<k<⅚$, it is the case that $\Delta_{0}^{s}\left( A_{\max} \right)⋚\Delta_{1}^{w}\left( A_{\min} \right)$ if $h⋚1/[6\left( 1-k \right)]$.


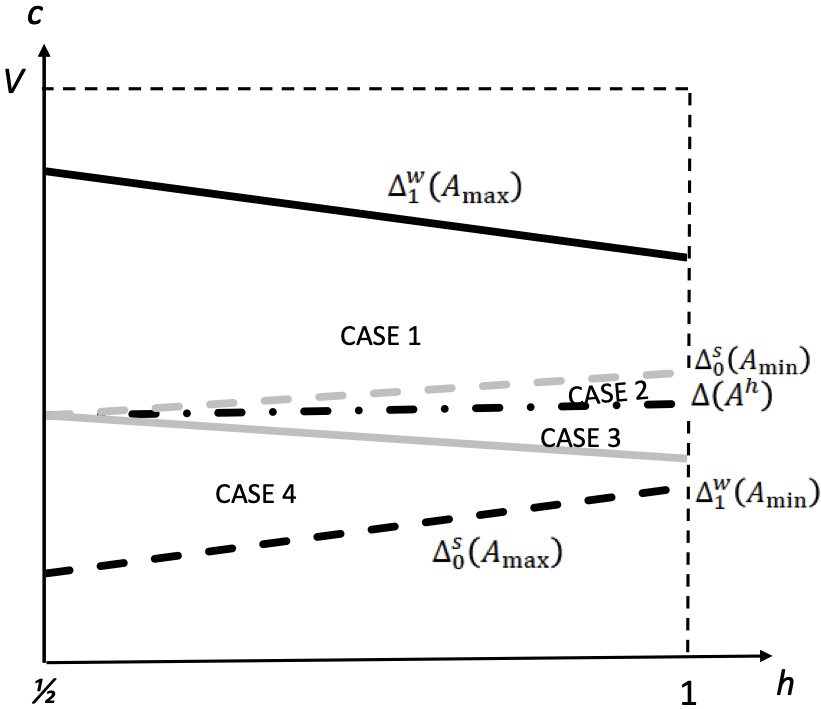


**Fig. S4. Defence variant with heterogeneous capability to contribute to defence**, where adversity is measured by the number of random attacks ($a=A$). Critical added benefits as a function of heterogeneity $h$. In line with the general version of the Results in Section 2, the different cases for the effect of higher adversity (see Table 3 in the report) are indicated: Case 1: symmetric common-enemy effect; Case 2: first asymmetric deterrence effect, then symmetric common-enemy effect; Case 3: first asymmetric common-enemy effect, then symmetric deterrence effect; Case 4: symmetric deterrence effect. The case represented is one where the underlying degree of complementarity $k$ is large, with $k>⅚$ (in particular, $k=0.85$).

As it is always the case that ${\Delta_{1}^{w}\left( A_{\max} \right)>\Delta}_{0}^{s}\left( A_{\max} \right)$, the case of large heterogeneity in the general version of the results in Section 2 is not relevant here; this means that the scenario in Figure 1(d) of the report, with only Cases 5 and 6 of Table 3 in the report occurring, is never relevant. Moreover, with large cooperation costs, it is always the case that $\Delta_{0}^{s}\left( A_{\min} \right)<\Delta_{1}^{w}\left( A_{\max} \right)$, so that furthermore the case of the upper range of small heterogeneity is never relevant; this means that Case 5 in Table 3 of the report never occurs in the defence variant with heterogeneous capabilities, and that the part of Figure 1(c) in the report with large cooperation costs is never relevant here. With small cooperation costs, when $⅚<k<1$ (represented in Figure S4), it is also always the case that $\Delta_{0}^{s}\left( A_{\max} \right)<\Delta_{1}^{w}\left( A_{\min} \right)$, so that the case of the upper range of small heterogeneity is never relevant here either, meaning that Case 6 of Table 3 in the report then also never occurs, so that the scenario in Figure 1(c) in the report is not relevant overall. With small cooperation costs, when instead $¾<k<⅚$, both the upper and the lower range of small heterogeneity is relevant, with Cases 3 and 4 of Table 3 in the report relevant for the lower range of small heterogeneity (scenario in Figure 1(b) in the report), and with Cases 3 and 6 relevant for the upper range of small heterogeneity (scenario in Figure 1(c) in the report). The results are now summarised in the modified version of the general version of the results in Section 2 for this variant:

**Results (version for the defence variant with** **heterogeneous capacity to contribute to the public good):** incidence of cases in Table 3 in the report for the effect of higher adversity, as function of cooperation costs $c$ and of heterogeneity $h$:

I. $h<1$:

A. $c>\frac{2k-1}{2[k-2\left( 1-k \right)h]}V[1-2\left( 1-k \right)h]$ (large $c$):

(1) (i) $0.5V[1-2\left( 1-k \right)(1-h)]<c<V\left[ 1-2\left( 1-k \right)h \right]$ (upper range of large $c$): Case 1;

(ii) $\frac{2k-1}{2[k-2\left( 1-k \right)h]}V\left[ 1-2\left( 1-k \right)h \right]<c<0.5V[1-2\left( 1-k \right)(1-h)]$ (lower range of large $c$): Case 2.

B. $c<\frac{2k-1}{2[k-2\left( 1-k \right)h]}V[1-2\left( 1-k \right)h]$ (small $c$):

(1) $h<1$ when $⅚<k<1$, or $h<1/[6(1-k)]$ when $¾<k<⅚$ (lower range of small $h$):

(i) $0.5V\left[ 1-2\left( 1-k \right)h \right]<c<\frac{2k-1}{2[k-2\left( 1-k \right)h]}V[1-2\left( 1-k \right)h]$ (upper range of small $c$): Case 3;

(ii) $2\left( 1- k \right)hV<c<0.5V\left[ 1-2\left( 1-k \right)h \right]$ (lower range of small $c$): Case 4.

(2) $1/[6(1-k)]<h<1$ when $¾<k<⅚$: (upper range of small $h$):

(i) $2\left( 1- k \right)hV<c<\frac{2k-1}{2[k-2\left( 1-k \right)h]}V[1-2\left( 1-k \right)h]$ (upper range of small $c$): Case 3;

(ii) $0.5V\left[ 1-2\left( 1-k \right)h \right]<2\left( 1- k \right)hV$ (lower range of small $c$): Case 3*.

For large complementarity ($k>⅚$, represented in Figure S4), the effect of a larger number of attacks is the same as the effect of increased complementarity in the variant with production of a public good. As a higher number of attacks has the same effect as an increase the degree of complementarity in the production variant, and as the fixed degree of complementarity is large, a high number of attacks means that the players’ efforts are to a large extent complementary, which neutralises the effects of heterogeneity (meaning that Cases 5 and 6 in Table 3 of the report never occur). Yet, when complementarity is not large ($¾<k<⅚$), even for a large number of attacks, players’ efforts are not fully pivotal, explaining why heterogeneity can have an effect, even when the number of attacks is large. The reason why Case 5 in Table 3 of the report still never occurs, is that without full complementarity, $b^{s}\left( D,D \right)=b^{w}\left( D,D \right)$ does not equal zero. For this reason, unlike what is the case in the scenario in Figure 1(a) of the report, for minimal heterogeneity, $\Delta_{1}^{w}$ is relatively steep and $\Delta_{0}^{s}$ relatively flat. Increases in heterogeneity, which shift $\Delta_{1}^{w}$ down and $\Delta_{0}^{s}$ up, can now be such that the relation between $\Delta_{1}^{w}\left( A_{\min} \right)$ and $\Delta_{0}^{s}\left( A_{\max} \right)$ changes (meaning that Case 6 of Table 3 in the report becomes possible), but cannot be such that the relation between $\Delta_{0}^{s}\left( A_{\min} \right)$ and $\Delta_{1}^{w}\left( A_{\max} \right)$ changes (meaning that Case 5 is not possible).

*4.2 Heterogeneity in value obtained from the public good*

We now again assume that all players contribute the same value to the public good $(b^{i}\left( C,D \right)=b^{i}\left( D,C \right)$ for $i=s,w$), but obtain different shares from the public good. It follows that $b^{S}\left( C,C \right)=h2V$, $b^{w}\left( C,C \right)=(1-h)2V$, $b^{s}\left( D,D \right)=h2\frac{2}{2^{A}}\left( 1-k \right)V$, $b^{w}\left( D,D \right)=(1-h)2\frac{2}{2^{A}}\left( 1-k \right)V$. Also, $b^{s}\left( C,D \right)=b^{s}\left( D,C \right)=\frac{1}{2^{A}}h2V+\left[ 1-\frac{1}{2^{A}} \right]h2\left( 1- k \right)V$ , and $b^{w}\left( C,D \right)=b^{w}\left( D,C \right)=\frac{1}{2^{A}}\left( 1-h \right)2V+\left[ 1-\frac{1}{2^{A}} \right](1-h)2\left( 1- k \right)V$. One can now check that $\Delta_{0}^{s}$ and $\Delta_{1}^{w}$ equal:

$\Delta_{0}^{s}=h2\left\{ \frac{1}{2^{A}}(2k-1)V+\left[ 1-\frac{1}{2^{A}} \right]\left( 1- k \right)V \right\}$ (S31)

$\Delta_{1}^{w}=\left( 1-h \right)2\left[ 1-\frac{1}{2^{A}} \right]kV$ (S32)

It is the case that $\frac{{\partial\Delta}_{0}^{s}}{\partial A}<0$, so that we are in the framework set out of the general version of the results in Section 2, as long as $k>⅔$, which we here systematically assume. The critical values, which are represented in Figure S5, are this time:

$\Delta_{0}^{s}\left( A_{\min} \right)=hkV$ (S33)

$\Delta_{1}^{w}\left( A_{\min} \right)=\left( 1-h \right)kV$ (S34)

$\Delta_{0}^{s}\left( A_{\max} \right)=h2\left( 1- k \right)V$ (S35)

$\Delta_{1}^{w}\left( A_{\max} \right)=\left( 1-h \right)2kV$ (S36)

$\Delta\left( A^{h} \right)=(1-h)\frac{2h\left( 2k-1 \right)}{2h(k-1)+k}kV$, with $A^{h}=ln\left\{ \frac{2h\left( k-1 \right)+k}{k-h} \right\}/\ln(2)$

(S37)

For $h=½$, $\Delta_{0}^{s}\left( A_{\min} \right)=\Delta\left( A^{h} \right)=\Delta_{1}^{w}\left( A_{\min} \right)=½kV$, $\Delta_{1}^{w}\left( A_{\max} \right)=kV$, and $\Delta_{0}^{s}\left( A_{\max} \right)=\left( 1-k \right)V$, where $\Delta_{0}^{s}\left( A_{\max} \right)<\Delta_{1}^{w}\left( A_{\min} \right)=\Delta\left( A^{h} \right)=\Delta_{0}^{s}\left( A_{\min} \right)<\Delta_{1}^{w}\left( A_{\max} \right)$. For $h=k$, $\Delta_{1}^{w}\left( A_{\min} \right)=k\left( 1-k \right)V$,$\Delta_{0}^{s}\left( A_{\max} \right)=\Delta\left( A^{h} \right)=\Delta_{1}^{w}\left( A_{\max} \right)=2k\left( 1- k \right)V$, and $\Delta_{0}^{s}\left( A_{\min} \right)=k^{2}V$. Given that $k>⅔$, it is therefore the case for $h=k$ that$\Delta_{1}^{w}\left( A_{\min} \right)<\Delta_{0}^{s}\left( A_{\max} \right)=\Delta\left( A^{h} \right)=\Delta_{1}^{w}\left( A_{\max} \right)<\Delta_{0}^{s}\left( A_{\min} \right)$. For $h=1$,$\Delta_{0}^{s}\left( A_{\min} \right)=kV$, $\Delta_{1}^{w}\left( A_{\min} \right)=0$, $\Delta_{0}^{s}\left( A_{\max} \right)=2\left( 1- k \right)V$, $\Delta_{1}^{w}\left( A_{\max} \right)=0$, $\Delta\left( A^{h} \right)=0$. Given that $k>⅔$, it is therefore the case for $h=1$ that ${{\Delta_{1}^{w}\left( A_{\min} \right)=\Delta\left( A^{h} \right)=\Delta_{1}^{w}\left( A_{\max} \right)<\Delta}_{0}^{s}\left( A_{\max} \right)<\Delta}_{0}^{s}\left( A_{\min} \right)$.

As long as $h<1$, it is the case that $\Delta_{1}^{w}\left( A_{\max} \right)>\Delta_{1}^{w}\left( A_{\min} \right)$, where both are decreasing functions in $h$. Given that $k>⅔$, $\Delta_{0}^{s}\left( A_{\min} \right)$ lies everywhere above $\Delta_{0}^{s}\left( A_{\max} \right)$, where both are increasing functions of $h$. As long as $h>½$,$\Delta_{0}^{s}\left( A_{\min} \right)$ lies everywhere above $\Delta_{1}^{w}\left( A_{\min} \right)$. As long as $½<h<1$, $\Delta\left( A^{h} \right)$ lies strictly in between $\Delta_{0}^{s}\left( A_{\min} \right)$ and $\Delta_{1}^{w}\left( A_{\min} \right)$. Also, as long as $h>½$, $\Delta_{0}^{s}\left( A_{\min} \right)$ lies everywhere above $\Delta_{1}^{w}\left( A_{\min} \right).$ Finally, it can be checked that $\Delta_{1}^{w}\left( A_{\max} \right)=\left( 1-h \right)2kV⋛\Delta_{0}^{s}\left( A_{\min} \right)=hkV$ iff $h⋚⅔$; that $\Delta_{1}^{w}\left( A_{\min} \right)=\left( 1-h \right)kV{⋛\Delta}_{0}^{s}\left( A_{\max} \right)=h2\left( 1- k \right)V$ iff $h⋚k/(2-k)$; and that $\Delta_{1}^{w}\left( A_{\max} \right)=\left( 1-h \right)2kV⋛\Delta_{0}^{s}\left( A_{\max} \right)=h2\left( 1- k \right)V$ iff $h⋚k$.


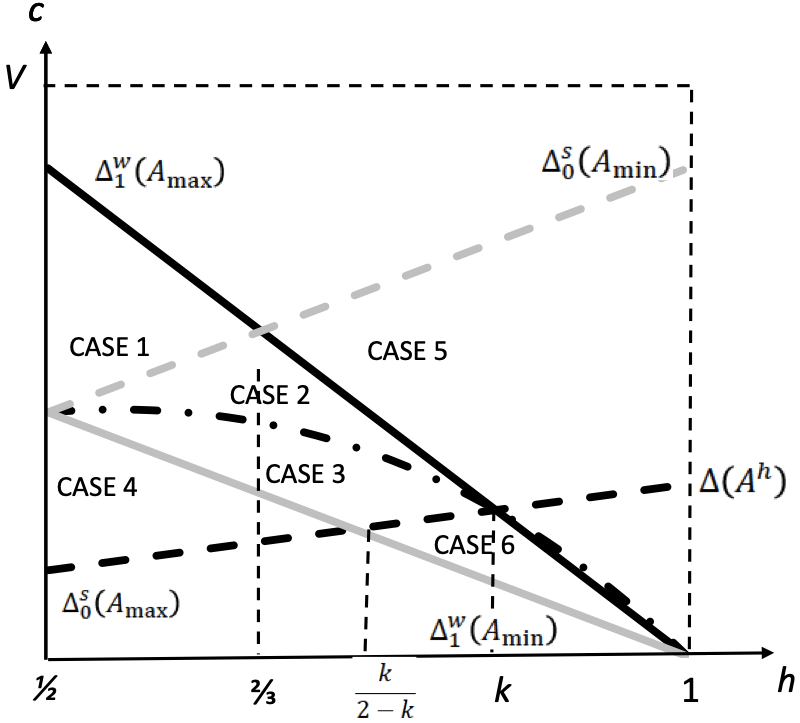


**Fig. S5. Defence variant with heterogeneous values obtained from the defended public good**, where adversity is measured by the number of random attacks ($a=A$). Critical added benefits as a function of heterogeneity $h$. In line with general version of the results in Section 2, the different cases for the effect of higher adversity (see Table 3 in the report) are indicated: Case 1: symmetric common-enemy effect; Case 2: first asymmetric deterrence effect, then symmetric common-enemy effect; Case 3: first asymmetric common-enemy effect, then symmetric deterrence effect; Case 4: symmetric deterrence effect; Case 5: asymmetric deterrence effect; Case 6: asymmetric common-enemy effect. The case represented is one where the underlying degree of complementarity $k$ is large, and meets $k>⅘$ (in particular, in the case represented $k=0.85$).

It follows that for sufficiently small heterogeneity, we obtain the scenario corresponding to Figure 1(b) in the main text (Cases 1, 2, 3 and 4 of Table 3 in the report all possible), and for sufficiently large heterogeneity, we obtain the scenario corresponding to Figure 1(d) in the report (only Cases 5 and 6 of Table 3 in the report possible). As $k>k/(2-k)$ as long as $k<1$, and as $k/(2-k)⋛⅔$ iff $k⋛⅘$, two cases are now possible. For $⅔<k<⅘$ (not represented in Figure S5), Cases 1 and 2 of Table 3 in the report can occur for large cooperation costs, while Cases 3 and 6 occur for small cooperation costs (meaning that Figure 1(b) in the report is the relevant scenario for large, and Figure 1(c) is the relevant scenario for small cooperation costs). For $k>⅘$ (represented in Figure S5), Cases 2 and 5 can occur for large cooperation costs, while Cases 3 and 4 occur for small cooperation costs (meaning that Figure 1(c) in the report is the relevant scenario for large cooperation costs, and Figure 1(b) is the relevant scenario for small cooperation costs). Note that in the limit case of full complementarity, Figure 1(d) in the main text is never relevant, and moreover Case 6 of Table 3 in the report never occurs; the case is then very similar to the effect of increased complementarity in the variant with production of a public good. Thus, Figure 1(c) in the main text becomes relevant for small cooperation costs, and Figure 1(d) becomes relevant overall, as long as $k$ is not maximal.

**Results (version for the** **defence variant with** **heterogeneous values obtained from the public good):** incidence of cases in Table 3 in the report for the effect of higher adversity, as function of cooperation costs $c$ and of absolute heterogeneity $h$:

I. $h<k$ (small $h$):

A. $c>(1-h)\frac{2h\left( 2k-1 \right)}{2h(k-1)+k}kV$ (large $c$):

(1) $h<⅔$ (lower range of small $h$):

(i) $hkV<c<\left( 1-h \right)2kV$ (upper range of large $c$): Case 1;

(ii) $\left( 1-h \right)\frac{2h\left( 2k-1 \right)}{2h\left( k-1 \right)+k}kV<c<hkV$ (lower range of large $c$): Case 2.

(2) $⅔<h<k$ (upper range of small $h$):

(i) $\left( 1-h \right)2kV<c<hkV$ (upper range of large $c$): Case 5;

(ii) $\left( 1-h \right)\frac{2h\left( 2k-1 \right)}{2h\left( k-1 \right)+k}kV<c<\left( 1-h \right)2kV$ (lower range of large $c$): Case 2.

B. $c<(1-h)\frac{2h\left( 2k-1 \right)}{2h(k-1)+k}kV$ (small $c$):

(1) $h<k/(2-k)$ (lower range of small $h$):

(i) $h2\left( 1- k \right)V<c<\left( 1-h \right)\frac{2h\left( 2k-1 \right)}{2h\left( k-1 \right)+k}kV$ (upper range of small $c$): Case 3;

(ii) $\left( 1-h \right)kV<c<h2\left( 1- k \right)V$ (lower range of small $c$): Case 4.

(2) $k/(2-k)<h<k$ (upper range of small $h$):

(i) $\left( 1-h \right)kV<c<\left( 1-h \right)\frac{2h\left( 2k-1 \right)}{2h\left( k-1 \right)+k}kV$ (upper range of small $c$): Case 3;

(ii) $h2\left( 1- k \right)V<c<\left( 1-h \right)kV$ (lower range of small $c$): Case 6.

II. $h>k$ (large $h$):

A. $hkV<c<h2\left( 1- k \right)V$ (large $c$): Case 5;

B. $\left( 1-h \right)kV<c<\left( 1-h \right)2kV$ (small $c$): Case 6.

These results are best understood by comparing to the production variant with heterogeneous values attached to the public good. When $k$ is maximal in the defence model ($k=1$), the effects of an increase in the degree of complementarity in the production model, and of an increase in the number of attacks in the defence variant, are fully equivalent, where heterogeneity does not have any effect on $\Delta_{0}^{s}$ in (S31) when the number of attacks becomes very large, explaining why Case 6 never becomes relevant. Yet, when $k<1$ in the defence model, even for a very large number of attacks, heterogeneity continues to have an impact on $\Delta_{0}^{s}$ in (S31). For this reason, as we increase heterogeneity, Case 6 in Table 3 of the report will also become relevant – even though just as in the model with production of a public good, the relevance of this case vanishes as heterogeneity reaches its maximum level.

*4.3 Heterogeneity in cooperation costs*

In the defence variant with heterogeneous costs, the added benefits are identical to those in (S22)-(S23), but cooperation costs differ in the same manner as in Section 3.3. Without loss of generality, in order to keep the analysis comparable to the other cases, we again consider adjusted added benefits, where added benefits are divided by the coefficient of the average costs.

$\Delta_{0}^{s}=1/[2(1-h)]\left\{ \frac{1}{2^{A}}(2k-1)V+\left[ 1-\frac{1}{2^{A}} \right]\left( 1- k \right)V \right\}$ (S38)

$\Delta_{1}^{w}=1/(2h)\left[ 1-\frac{1}{2^{A}} \right]kV$ (S39)

Just as for defence variant with vanishing heterogeneity, it is the case that $\frac{{\partial\Delta}_{0}^{s}}{\partial A}<0$ as long as $k>⅔$. Following the general version of the results in Section 2, the critical added benefits, represented in Figure S6, are this time (with $a=A$):

$\Delta_{0}^{s}\left( A_{\min} \right)=kV/[4(1-h)]$ (S40)

$\Delta_{1}^{w}\left( A_{\min} \right)=kV/(4h)$ (S41)

$\Delta_{0}^{s}\left( A_{\max} \right)=\left( 1- k \right)V/[2(1-h)]$ (S42)

$\Delta_{1}^{w}\left( A_{\max} \right)=kV/(2h)$ (S43)

$\Delta\left( A^{h} \right)=kV/(2h)\frac{h\left( 2k-1 \right)}{2h\left( k-1 \right)+k}$, with $A^{h}=ln\left\{ \frac{2h\left( k-1 \right)+k}{k-h} \right\}/\ln(2)$

(S45)


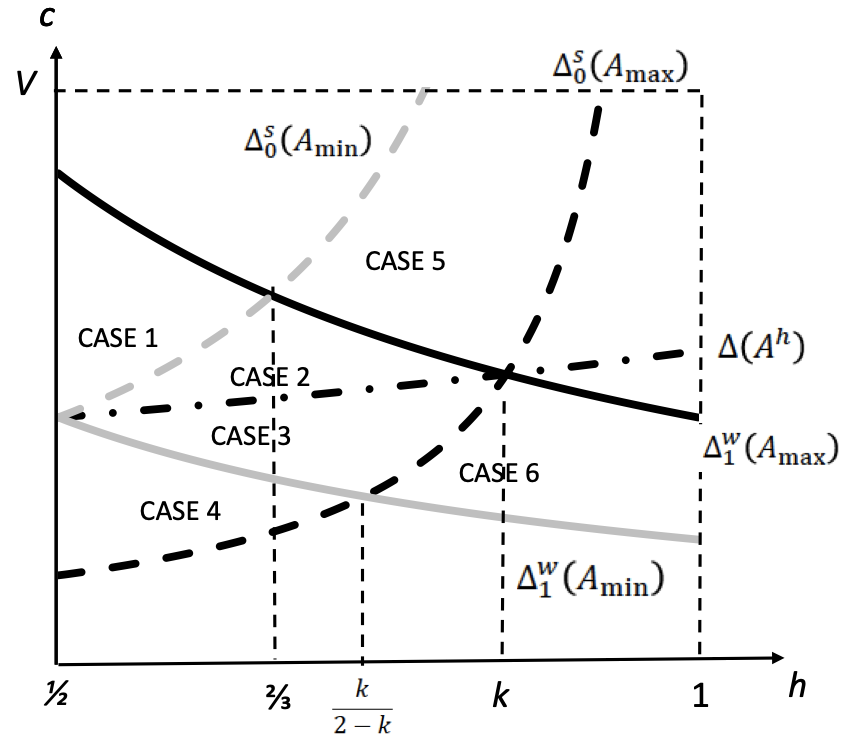


**Fig. S6.** Defence variant with heterogeneous cooperation costs, where adversity is measured by the number of random attacks ($a=A$). Critical added benefits as a function of heterogeneity $h$. In line with the general version of the results in Section 2, the different cases for the effect of higher adversity (see Table 3 in the report) are indicated: Case 1: symmetric common-enemy effect; Case 2: first asymmetric deterrence effect, then symmetric common-enemy effect; Case 3: first asymmetric common-enemy effect, then symmetric deterrence effect; Case 4: symmetric deterrence effect; Case 5: asymmetric deterrence effect; Case 6: asymmetric common-enemy effect. In the case represented, $k=0.85$.

For $h=½$, $\Delta_{0}^{s}\left( A_{\min} \right)=\Delta\left( A^{h} \right)=\Delta_{1}^{w}\left( A_{\min} \right)=½kV$, $\Delta_{1}^{w}\left( A_{\max} \right)=kV$, and $\Delta_{0}^{s}\left( A_{\max} \right)=\left( 1-k \right)V$, where $\Delta_{0}^{s}\left( A_{\max} \right)<\Delta_{1}^{w}\left( A_{\min} \right)=\Delta\left( A^{h} \right)=\Delta_{0}^{s}\left( A_{\min} \right)<\Delta_{1}^{w}\left( A_{\max} \right)$. For $h=k$, $\Delta_{1}^{w}\left( A_{\min} \right)=¼V$,$\Delta_{0}^{s}\left( A_{\max} \right)=\Delta\left( A^{h} \right)=\Delta_{1}^{w}\left( A_{\max} \right)=½V$, and $\Delta_{0}^{s}\left( A_{\min} \right)=kV/[4(1-k)]$. Given that $k>⅔$, it is therefore the case for $h=k$ that$\Delta_{1}^{w}\left( A_{\min} \right)<\Delta_{0}^{s}\left( A_{\max} \right)=\Delta\left( A^{h} \right)=\Delta_{1}^{w}\left( A_{\max} \right)<\Delta_{0}^{s}\left( A_{\min} \right) .$For $h=1$,$\Delta_{0}^{S}\left( A_{\min} \right)=+\infty$, $\Delta_{1}^{w}\left( A_{\min} \right)=¼kV$, $\Delta_{0}^{s}\left( A_{\max} \right)=+\infty$, $\Delta_{1}^{w}\left( A_{\max} \right)=½kV$, $\Delta\left( A^{h} \right)=0$. Given that $k>⅔$, it is therefore the case for $h=1$ that $\Delta_{1}^{w}\left( A_{\min} \right)<\Delta_{1}^{w}\left( A_{\max} \right)<\Delta\left( A^{h} \right)$.

It is the case that $\Delta_{1}^{w}\left( A_{\max} \right)>\Delta_{1}^{w}\left( A_{\min} \right)$, where both are convex decreasing functions in $h$. Given that $k>⅔$, $\Delta_{0}^{s}\left( A_{\min} \right)$ lies everywhere above $\Delta_{0}^{s}\left( A_{\max} \right)$, where both are convex increasing functions of $h$. As long as $h>½$,$\Delta_{0}^{s}\left( A_{\min} \right)$ lies everywhere above $\Delta_{1}^{w}\left( A_{\min} \right)$. As long as $h>½$, $\Delta\left( A^{a} \right)$ lies strictly in between $\Delta_{0}^{s}\left( A_{\min} \right)$ and $\Delta_{1}^{w}\left( A_{\min} \right).$ Finally, it can be checked that $\Delta_{1}^{w}\left( A_{\max} \right)=kV/(2h)⋛\Delta_{0}^{s}\left( A_{\min} \right)=kV/[4(1-h)]$ iff $h⋚⅔$; that $\Delta_{1}^{w}\left( A_{\min} \right)=kV/(4h){⋛\Delta}_{0}^{s}\left( A_{\max} \right)=\left( 1- k \right)V/[2(1-h)]$iff $h⋚k/(2-k)$; and that $\Delta_{1}^{w}\left( A_{\max} \right)=kV/(2h)⋛\Delta_{0}^{s}\left( A_{\max} \right)=\left( 1- k \right)V/[2(1-h)]$ iff $h⋚k$. It follows that qualitatively, the results are very similar to those for the variant with production of a public good and heterogeneous costs.

**Results (version for the** **defence variant** **with** **heterogeneous costs):** incidence of cases in Table 3 of the report for the effect of higher adversity, as function of cooperation $c$ costs and of heterogeneity $h$:

I. $h<k$ (small $h$):

A. $c>kV/(2h)\frac{h\left( 2k-1 \right)}{2h\left( k-1 \right)+k}$ (large $c$):

(1) $h<2/3$ (lower range of small $h$):

(i) $kV/[4(1-h)]<c<kV/(2h)$ (upper range of large $c$): Case 1;

(ii) $kV/(2h)\frac{h\left( 2k-1 \right)}{2h\left( k-1 \right)+k}V<c<kV/[4(1-h)]$ (lower range of large $c$): Case 2.

(2) $2/3<h<k$ (upper range of $h$):

(i) $kV/(2h)<c<kV/[4(1-h)]$ (upper range of large $c$): Case 5;

(ii) $kV/(2h)\frac{h\left( 2k-1 \right)}{2h\left( k-1 \right)+k}V<c<kV/(2h)$ (lower range of large $c$): Case 2.

B. $c<kV/(2h)\frac{h\left( 2k-1 \right)}{2h\left( k-1 \right)+k}$ (small $c$):

(1) $h<k/(2-k)$ (lower range of small $h$):

(i) $kV/(4h)<c<kV/(2h)\frac{h\left( 2k-1 \right)}{2h\left( k-1 \right)+k}$ (upper range of small $c$): Case 3;

(ii) $\left( 1- k \right)V/[2(1-h)]<c<kV/(4h)$ (lower range of small $c$): Case 4.

(2) $k/(2-k)<h<k$ (upper range of small $h$):

(i) $\left( 1- k \right)V/[2(1-h)]<c<kV/(2h)\frac{h\left( 2k-1 \right)}{2h\left( k-1 \right)+k}V$ (upper range of small $c$): Case 3;

(ii) $kV/(4h) <c<\left( 1- k \right)V/[2(1-h)]$ (lower range of small $c$): Case 6.

II. $h>k$ (large $h$):

A. $\left( 1- k \right)V/[2(1-h)]<c<kV/[4(1-h)]$ (large $c$): Case 5;

B. $kV/(4h)<c<kV/(2h)$(small $c$): Case 6.

Just as was the case in the production variant of the model, the results for heterogeneous shares obtained from the defended public good, and for heterogeneous costs, are similar. The difference with the results for production variant are again that Case 6 of Table 3 in the report does not vanish for large heterogeneity.

**5. By-product mutualism with a private good that produces a by-product benefit**

We consider here an alternative model of by-product mutualism where a player who cooperates produces a private good to himself, which creates by-product benefits to the other players in his group. Mesterton-Gibbons and Dugatkin (1992) refer to the example of house sparrows producing chirrups when having found a large food source (Elgar, 1986). While this means attracting other sparrows to the food source (by-product benefit to the other sparrows), it also means decreasing the impact of predation (private good to the cooperating sparrow). Consider now a highly stylised case where two sparrows can each call the other sparrow when finding a large food source (*C*), or not call the other sparrow (*D*). Then denote as $\mathcal{B}$ the by-product benefit produced for the considered sparrow of being called by the other sparrow to its food source; note that this by-product benefit does not depend on whether the considered sparrow cooperates or defects itself. Also, denote as $\mathcal{C}$ the net cost of producing a chirrup, which includes the cost of having to share the food source, corrected by the benefit obtained by reducing the impact of predation (hence the term net costs), where the net cost is negative if this benefit exceeds the cost of food sharing (applying the net cost concept to the public good game in the main text, the negative of the added benefit of cooperating can be considered as a net cost). This net cost does not depend on whether or not the other sparrow is cooperating (this is the so-called “equal gains from switching property”, Nowak and Sigmund 1990). The game is equivalent to a donor-recipient game, where both players obtain $\mathcal{B-C}$ if both players cooperate, where the cooperating player obtains $\mathcal{-C}$ and the defecting player $\mathcal{B}$ if one player cooperates and the other defects, and both players obtain 0 if both players defect. The form of the ESS is now purely determined by whether $\mathcal{C}$ is positive or negative, and the size of the by-product benefit does not play any role. As represented in Figure S.1(a), where $-\mathcal{C}$ is represented along the Y-axis, as the risk of predation is increased, the added benefit of reducing the impact of predation increases, and with it $-\mathcal{C}$ increases. The game may now change from a Prisoner’s Dilemma into a Harmony Game, in line with the common-enemy hypothesis of by-product mutualism. The underlying mechanism is again what Mesterton-Gibbons and Dugatkin (1992) call the boomerang effect: a harsher environment (here in the form of a higher risk of predation) causes a defector to become the victim of its own defection.

(a) Homogeneous model (b) Heterogeneous model

$$-\mathcal{C}$$

$$-\mathcal{C}$$

$-\mathcal{C}^{s}$

$$-\mathcal{C}$$

$-\mathcal{C}^{w}$

0

0

*a*

*a*

HG

FR

PD

HG

PD

**Fig. S1.** (a) Homogeneous and (b) heterogeneous private-good model. Net cost of cooperating ($-\mathcal{C}$) as a function of adversity $a$. In the heterogeneous model, the net cost is higher for the weak type ($\mathcal{C}^{w}$) than for the strong type ($\mathcal{C}^{s}$). As a function of the sign of the added payoffs, the game is either a Prisoner’s Dilemma (PD) or a Harmony Game (HG); with heterogeneity, for intermediate degrees of adversity the game may additionally be a Free-Rider game (FR), where only the strong player cooperates. In both the homogeneous and the heterogeneous model, the only possible prediction for the effect of higher adversity on the probability of cooperation fits the common-enemy hypothesis.

Another example fitting the model of private-good production with a by-product benefit may be fiddler crabs (Detto et al. 2010), which have been observed to assist neighbouring crabs to defend their territories against intruders. The benefit part of the net cost of cooperating this time is that the individual crab in this way avoids the risk of having a new and stronger neighbour. The degree of adversity here may be measured by the probability that the intruder is stronger. Further examples fitting such a scenario may include communally breeding birds, where non-related fledglings are fed to avoid that their begging attracts predators to own fledglings (Caraco and Brown 1986), where the risk of predation again measures adversity; alternatively, insects that avoid laying an egg on a host that is already parasitised, because this reduces the probability that they lay an egg on a host that they already parasitised themselves, where adversity is measured by the survival probability of a solitary egg relative to a paired egg (Mesterton-Gibbons, 1991). In all these examples, cooperating is done out of ordinary self-interest; the individual player who cooperates is really producing a private good to himself (protection of predators, avoidance of a potentially strong new neighbour) and producing this private good happens to create a by-product benefit to other players (access to a food source, defence against an intruder).

It is clear that in this model of by-product mutualism, heterogeneity between the players does not fundamentally change the results. As represented in Figure S1(b), the only change is that the net cost now differs between strong and weak players. Thus, while it continues to be the case that for low adversity all players defect, and for high adversity all players cooperate, for intermediate adversity, only strong players may act.

**6. Groups with more than two players**

Consider an $n$-player variant of the two-player games treated in the main text. Following the structure of the paper, we first provide a general framework, and then specific cases. Denote by $x$, with $1\leq x\leq n$, the rank of a player, where the player with rank 1 is the strongest player, the player with rank 2 the one-but-strongest player, and so on. We continue to assume that each player can cooperate or defect, where as a function of the number of cooperating players, each player either obtains the same value of a public good, or obtains a share of this value. It continues to be the case that if all players defect value 0 is produced, and if all players cooperate value $V$ is produced.

For any given number of players that currently cooperate, among the players that defect, the player with the largest added payoff of cooperating is the player with the lowest $x$ (i.e. the most highly ranked player) (where the added payoff contains both the added benefit of cooperating, and cooperation costs). Thus, the relevant added payoff of cooperating with 1 player (rather than with 0 players) to consider is the added payoff of the player with rank 1, the relevant added payoff to consider of cooperating with 2 players (rather than with 1 player) is the added benefit of the player with rank 2, and so on. Consider in general the added payoff of cooperating rather than defecting with $x$ players to the player with rank $x$, as a function of $x$, denoted as $\delta_{x-1}^{x}=\Delta_{x-1}^{x}-c^{x}$, where the superscript refers to the rank, and the subscript to the number of players cooperating before the considered player joins in cooperating (where $\Delta_{x-1}^{x}$ denotes the added benefit, and $c^{x}$ the cooperation costs of this player).

Adversity means that as we approach the limit case of vanishing heterogeneity, where for any given number of cooperating players the added payoffs of cooperating do not differ across players, $\delta_{x-1}^{x}$ increases in $x$, meaning that the last cooperating players contribute more to the value of the public good than the first cooperating players. Whatever the level of heterogeneity, higher adversity means making $\delta_{x-1}^{x}$ larger for any rank smaller than some rank $y$, and smaller for any rank larger than this rank $y$, reflecting the idea that higher adversity increases the added payoffs of cooperating when many players are already cooperating, and reduces the added payoffs of cooperating when few players are cooperating.

Heterogeneity means that as we approach the limit case with minimal adversity, $\delta_{x-1}^{x}$ decreases in $x$, where a player’s added payoff is larger the stronger he is. In general, whatever the degree of adversity, increasing the degree of heterogeneity means increasing $\delta_{x-1}^{x}$ for any rank smaller than some rank $z$, and decreasing $\delta_{x-1}^{x}$ for any rank larger than some rank $z$, reflecting the idea that increasing the level of heterogeneity gives stronger players more incentives to cooperate, and weaker players fewer incentives to cooperate.^[[2]](#footnote-2)^

Heterogeneity is relatively small when $\delta_{x-1}^{x}$ increases in $x$ (in which case adversity is the dominant factor, which means that the added payoff of cooperating is higher the more players cooperate), and heterogeneity is relatively large when $\delta_{x-1}^{x}$ decreases in $x$. In Figure S7, the lines in the top part sketch $\delta_{x-1}^{x}$ for relatively small heterogeneity, and in the bottom part sketch $\delta_{x-1}^{x}$ for relatively large heterogeneity (where for ease of exposition, $x$ is considered as a real number rather than an integer). The solid lines represent $\delta_{x-1}^{x}$ for an initial degree of adversity, where $x_{1}$ is the rank around which $\delta_{x-1}^{x}$ changes sign. With relatively weak heterogeneity, $\delta_{x-1}^{x}⋛0$ for $x⋛x_{1}$, meaning that the game has Stag Hunt features, where for a sufficient number of cooperating players, each player is better off cooperating, and for an insufficient number of cooperating players, each player is better off defecting. An increase in adversity tilts $\delta_{x-1}^{x}$ counter-clockwise, and results in the new $\delta_{x-1}^{x}$ represented by a dashed line. When cooperation costs are large, the position of $\delta_{x-1}^{x}$ is low, and the rank around which $\delta_{x-1}^{x}$ changes sign decreases to $x_{2}$. This means that a lower number of cooperating players is needed to make cooperating a best response, and suggests an increase in the basin of attraction of the joint cooperation ESS, corresponding to a symmetric common-enemy effect.^[[3]](#footnote-3)^ When cooperation costs are instead small, the position of $\delta_{x-1}^{x}$ is high, and the rank around which $\delta_{x-1}^{x}$ changes sign increases to $x_{2}$. A higher number of cooperating players is now needed to make cooperating a best response, which suggests a decrease in the basin of attraction of the joint cooperation ESS, corresponding to a symmetric deterrence effect.

With relatively large heterogeneity, $\delta_{x-1}^{x}⋛0$ for $x⋚x_{1}$, meaning that the game has Free-Rider game features, where in the unique ESS only the strongest players cooperate. An increase in adversity again tilts $\delta_{x-1}^{x}$ counter-clockwise, resulting in a new $\delta_{x-1}^{x}$ sketched a dashed line. For large cooperation costs, the position of $\delta_{x-1}^{x}$ is low, and the rank around which $\delta_{x-1}^{x}$ changes sign this time decreases to $x_{2}$. This means that in the unique ESS, the weakest players that were previously cooperating switch to defecting instead, corresponding to an asymmetric deterrence effect. When cooperation costs are instead small, the position of $\delta_{x-1}^{x}$ is again high, and the rank around which $\delta_{x-1}^{x}$ changes sign increases to $x_{2}$. This means that in the unique ESS, the strongest players that were previously defecting switch to cooperating instead, corresponding to an asymmetric common-enemy effect.

We now apply this general framework for multi-player games to the defence variant with heterogeneous cooperation costs, where we assume that complementarity is maximal. We choose the defence variant to avoid having to model a specific impact function relating players efforts to a value of the public good, such as the CES impact function (see e.g. Gravilets (2016)), which would further complicate the analysis. We assume full complementarity, because otherwise it matters how many different defecting players are attacked. Finally, we choose heterogeneous cooperation costs because in this way, the number of attacks (which acts as a measure of adversity) impacts upon the added benefits, whereas heterogeneity affects cooperation costs.

Under these assumptions, when $y$ players cooperate, this means that with probability ${(y/n)}^{A}$, all players obtain benefit $V$, whereas with the complementary probability, they all obtain benefit 0. It follows that $\Delta_{y-1}=V\{{[y/n]}^{A}-{[(y-1)/n]}^{A}\}$. This implies that $\Delta_{y-1}$ is flat in $y$ for $A$ fixed at $A=1$, and is increasing in $y$ for $A$ fixed at $A>1$. Moreover, for fixed $y$, a level of $y$ equal to $y_{0}$ exists such that $\Delta_{y-1}$ decreases in $A$ for $y<y_{0}$, and increases in $A$ for $y>y_{0}$.

(a) Relatively weak heterogeneity, large cooperation (b) Relatively weak heterogeneity, small cooperation

costs: symmetric common-enemy effect costs: symmetric deterrence effect

$$\delta_{x-1}^{x}(x)$$

$$\delta_{x-1}^{x}(x)$$

$$x_{2}$$

$$x_{1}$$

0

0

$$x_{1}$$

$$x_{2}$$

$$x$$

$$x$$

(c) Relatively strong heterogeneity, large cooperation (d) Relatively strong heterogeneity, small cooperation

costs: asymmetric deterrence effect costs: asymmetric common-enemy effect

$$\delta_{x-1}^{x}(x)$$

$$\delta_{x-1}^{x}(x)$$

$$x_{1}$$

$$x_{2}$$

$$x_{2}$$

$$x_{1}$$

0

0

$$x$$

$$x$$

**Fig. S7.** Added payoff of cooperating with $x$ players to the player with rank $x$, as a function of $x$, denoted $\delta_{x-1}^{x}$, where the lower $x$, the stronger the player. The solid line sketches $\delta_{x-1}^{x}$ for an initial degree of adversity, the dashed line sketches $\delta_{x-1}^{x}$ for higher adversity. In the top part of the figure, relative heterogeneity is weak, and in the bottom part it is strong; in the left figures, cooperation costs are large, and in the right figures they are small. $x_{1}$ and $x_{2}$ denote ranks around which $\delta_{x-1}^{x}$ changes sign. Each time, it is indicated which effect defined in Table 3 of the report applies.

As a function of the rank $x$ of a player, we assume that her cooperation costs equal $c^{x}={\{x}^{\gamma}/[\sum_{i=1}^{n} i^{\gamma}]\}(nc)$. In this manner, average cooperation costs equal $c$. The parameter $\gamma$ now serves as a measure of heterogeneity, where heterogeneity is minimal for $\gamma$ approaching zero (where each player has cooperation costs $c$), and increases as $\gamma$ is increased (where cooperation costs of the weakest player approach $nc$). $c^{x}$ is increasing in $x$, and the effect of an increase in $\gamma$ is always to increase the cooperation costs of a range of weaker players, and decrease the cooperation costs of a range of stronger players. If follows that

$\delta_{x-1}^{x}={V\{[x/n]}^{A}-{[(x-1)/n]}^{A}\}-{\{x}^{\gamma}/[\sum_{i=1}^{n} i^{\gamma}]\}(nc)$

Tables S1 and S2 represent the values of $\delta_{x-1}^{x}$ for the case where $n=5, V=1.2$. In Table S1, a case with relatively weak heterogeneity is represented, where $\gamma=0.1$. Higher adversity in the form of an increase from two attacks (*A* = 2) to three attacks (*A* = 3) is considered, both for large average cooperation costs (*c* = 0.33) and for small average cooperation costs (*c* = 0.1). In all cases, $\delta_{x-1}^{x}$ is negative for a range of small $x$ and positive for a range of large $x$, meaning that the game each time has Stag Hunt features. The grey numbers indicate $x$ such that $\delta_{x-1}^{x}$ is positive. For large average cooperation costs, the increase in adversity from *A* = 2 to *A* = 3 means that the added benefit of cooperating is larger than zero when 3 instead of 4 players are currently cooperating, suggesting an increase in the basin of attraction of the joint cooperation equilibrium, or a symmetric common-enemy effect. For small average cooperation costs, the increase in adversity from *A* = 2 to *A* = 3 implies that the added benefit of cooperating is larger than zero when 2 instead of 1 players are currently cooperating, suggesting a decrease in the basin of attraction of the joint cooperation equilibrium, or a symmetric deterrence effect.

**Table S1.** Multi-player example of the defence variant with heterogeneous costs (see the Methodology section), case of weak relative heterogeneity, where in (1), $n=5, V=1.2$, $\gamma=0.1$. Added payoff $\delta_{x-1}^{x}$ of cooperating with $x$ players to the player with rank $x$, as a function of $x$. For large average cooperation costs (*c* = 0.33), a symmetric common-enemy effect occurs, and for small average cooperation costs (*c* = 0.1), a symmetric deterrence effect (see Table 3 in the report).

| *c* = 0.33 | | | | *c* = 0.1 | | | |
| --- | --- | --- | --- | --- | --- | --- | --- |
| *A* = 2 | | *A* = 3 | | *A* = 2 | | *A* = 3 | |
| *x* | $\delta_{x-1}^{x}$ | *x* | $\delta_{x-1}^{x}$ | *x* | $\delta_{x-1}^{x}$ | *x* | $\delta_{x-1}^{x}$ |
| 1 | -0.251 | 1 | -0.290 | 1 | -0.043 | 1 | -0.081 |
| 2 | -0.177 | 2 | -0.254 | 2 | 0.047 | 2 | -0.030 |
| 3 | -0.094 | 3 | -0.152 | 3 | 0.139 | 3 | 0.081 |
| 4 | -0.008 | 4 | 0.011 | 4 | 0.232 | 4 | 0.251 |
| 5 | 0.080 | 5 | 0.234 | 5 | 0.325 | 5 | 0.479 |

Table S2 considers a case with relatively strong heterogeneity, where $\gamma=2$. This time, higher adversity in the form of an increase from one attack (*A* = 1) to two attacks (*A* = 2) is considered, for large average cooperation costs (*c* = 0.4) and for small average cooperation costs (*c* = 0.2). In all cases, $\delta_{x-1}^{x}$ is positive for a range of small $x$ and negative for a range of large $x$, so that the game each time has Free-Rider game features. The gray numbers again indicate $x$ such that $\delta_{x-1}^{x}$ is positive. For large average cooperation costs, the increase in adversity from *A* = 1 to *A* = 2 means that only the strongest player is better off cooperating, rather than both the strongest and the one-but strongest player, implying an asymmetric deterrence effect. For small average cooperation costs, the increase in adversity from *A* = 1 to *A* = 2 implies that all but the weakest player is better off cooperating, rather than all but the weakest and the one-but-weakest player, implying an asymmetric common-enemy effect.

**Table S2.** Multi-player example of the defence variant with heterogeneous costs (see the Methodology section), case of strong relative heterogeneity, where in (B.1), $n=5, V=1.2$, $\gamma=2$. Added payoff $\delta_{x-1}^{x}$ of cooperating with $x$ players to the player with rank $x$, as a function of $x$. For large average cooperation costs (*c* = 0.4), an asymmetric deterrence effect occurs, and for small average cooperation costs (*c* = 0.2), an asymmetric common-enemy effect (see Definition 2).

| *c* = 0.4 | | | | *c* = 0.2 | | | |
| --- | --- | --- | --- | --- | --- | --- | --- |
| *A* = 1 | | *A* = 2 | | *A* = 1 | | *A* = 2 | |
| *x* | $\delta_{x-1}^{x}$ | *x* | $\delta_{x-1}^{x}$ | *x* | $\delta_{x-1}^{x}$ | *x* | $\delta_{x-1}^{x}$ |
| 1 | 0.204 | 1 | 0.012 | 1 | 0.222 | 1 | 0.030 |
| 2 | 0.095 | 2 | -0.001 | 2 | 0.167 | 2 | 0.071 |
| 3 | -0.087 | 3 | -0.087 | 3 | 0.076 | 3 | 0.076 |
| 4 | -0.342 | 4 | -0.246 | 4 | -0.051 | 4 | 0.045 |
| 5 | -0.669 | 5 | -0.477 | 5 | -0.215 | 5 | -0.023 |

We end by noting that several complicating factors arise for multi-player heterogeneous games, which are not clear from the numerical examples above. First, because the number of attacks can only take on discrete values, the effect of higher adversity is not marginal, and for this reason there may be a switch from a decreasing to an increasing $\delta_{x-1}^{x}$ as $A$ is increased by one unit. Second, while the benefit and the cost part of $\delta_{x-1}^{x}$ are each monotonic in $x$, this does not mean that $\delta_{x-1}^{x}$ is always monotonic; the transition between increasing $\delta_{x-1}^{x}$ and decreasing $\delta_{x-1}^{x}$ is not always as clear-cut as in Tables S1 and S2. Third, as shown in De Jaegher (2017), with multiple players, the critical costs that separate the cases of large and small cooperation costs, are themselves a function of adversity. For this reason, higher adversity can have a non-monotonic effect even in the symmetric model. Yet, locally, for small changes in the degree of adversity, the distinction between small and large cooperation costs continues to make sense.

**References**

De Jaegher K., Harsh environments and the evolution of multi-player cooperation. *Theor. Pop. Biol.* **113**, 1–12 (2017).

De Jaegher, K. & Hoyer, B., By-product mutualism and the ambiguous effects of harsher environments: a game-theoretic model. *J. Theor. Biol.* **393**, 82–97 (2016)

Packer C. & Ruttan L. The evolution of cooperative hunting. *Am. Nat.* **132**, 159–198 (1988).

Detto, T., Jennions, M.D., & Backwell, P.R.Y. When and why do territorial coalitions occur? Experimental evidence from a fiddler crab. *Am. Nat.* **175**, E119–E125 (2010).

Dionisio, F. & Gordo, I. The tragedy of the commons, the public goods dilemma, and the meaning of rivalry and excludability in evolutionary biology. *Evol. Ecol. Res.* **8**, 321–332 (2006).

Elgar, M.A. House sparrows establish foraging flocks by giving chirrup calls if the resources are divisible. *Anim. Behav.* **34**, 169–174 (1986).

Gravilets, S. Collective action problem in heterogeneous groups. *Phil. Trans. R. Soc. B.* **370**, 1–17 (2015).

Hauert, C., Michor, F., Nowak, M.A. & Doebeli M. Synergy and discounting in social dilemmas. *J .Theor. Biol.* **239**, 195–202 (2006).

Heinsohn R, Packer C. Complex cooperative strategies in group-territorial African lions. Science. 1995; 269:1260–1262.

Jolivet P, Vasconcellos-Neto J, Weinstein P. Cycloalexy: a new concept in the larval defense of insects. Insect. Mundi. 1990; 4:133–141.

Mesterton-Gibbons, M.. An escape from ‘the prisoner’s dilemma’. J. Math Biol. 1991; 29:251–269.

Nowak, M.A., Sigmund, K.. The evolution of stochastic strategies in the prisoner’s dilemma. Acta Applicandae Mathematicae 1990; 20:247–265.

Ray, D., Baland, J.-M., Dagnelie, O.. Inequality and inefficiency in joint projects. Econ. J. 2007; 117:922–935.

Sugden, R. *The Economics of Rights, Cooperation and Welfare.* (Blackwell, 1986).

1. The degree of complementarity (and with it the degree of adversity) can be further reduced below ½ (De Jaegher and Hoyer, 2016). In this case, a single cooperating player may suffice to obtain nearly the maximal value of the public good (a single predator may be able to catch a prey from which a group benefits; a single vigilant player may suffice to defend a common territory). The game then becomes a so-called Snowdrift game (Sugden, 1986), with a mixed-population evolutionarily stable strategy where a fraction of the players cooperate. Since, just as is the case with large heterogeneity, the added benefit of cooperating alone is now larger than the added benefit of cooperating jointly, the homogeneous case for $k<½$ leads to similar results as for the case with large heterogeneity the general version of the results in Section 2 (Case II), with a common-enemy effect for small cooperation costs, and a deterrence effect for large cooperation costs. Because of this fact, when heterogeneity is added to cases where $k<½$ , the incidence of the common-enemy and deterrence effects does not change. It is because heterogeneity does not qualitatively affect the results here, that we do not consider cases with $k<½$. [↑](#footnote-ref-1)
2. As heterogeneity and the degree of complementarity have opposite effects, the analysis can also be used to analyze the effect of changes in heterogeneity, for fixed levels of the degree of complementarity. Ray et al. (2007) undertake such an analysis, and find that the sign of the effect of heterogeneity depends on the degree of complementarity, which is in line with our result that the sign of the effect of the degree of complementarity, depends on the degree of heterogeneity. [↑](#footnote-ref-2)
3. The size of the basin of attraction measures the probability that joint cooperation evolves if each initial population state is equally likely. The common-enemy effect is less pronounced if initial population states with defection are more likely, but is maintained as long as each population state occurs with positive probability. [↑](#footnote-ref-3)
